# Persistence and Adaptation in Immunity: T Cells Balance the Extent and Thoroughness of Search

**DOI:** 10.1371/journal.pcbi.1004818

**Published:** 2016-03-18

**Authors:** G. Matthew Fricke, Kenneth A. Letendre, Melanie E. Moses, Judy L. Cannon

**Affiliations:** 1 Department of Computer Science, The University of New Mexico, Albuquerque, New Mexico, United States of America; 2 Department of Biology, The University of New Mexico, Albuquerque, New Mexico, United States of America; 3 External Faculty, Santa Fe Institute, Santa Fe, New Mexico, United States of America; 4 Department of Molecular Genetics and Microbiology, The University of New Mexico School of Medicine, Albuquerque, New Mexico, United States of America; 5 Department of Pathology, The University of New Mexico School of Medicine, Albuquerque, New Mexico, United States of America; National Institutes of Health, UNITED STATES

## Abstract

Effective search strategies have evolved in many biological systems, including the immune system. T cells are key effectors of the immune response, required for clearance of pathogenic infection. T cell activation requires that T cells encounter antigen-bearing dendritic cells within lymph nodes, thus, T cell search patterns within lymph nodes may be a crucial determinant of how quickly a T cell immune response can be initiated. Previous work suggests that T cell motion in the lymph node is similar to a Brownian random walk, however, no detailed analysis has definitively shown whether T cell movement is consistent with Brownian motion. Here, we provide a precise description of T cell motility in lymph nodes and a computational model that demonstrates how motility impacts T cell search efficiency. We find that both Brownian and Lévy walks fail to capture the complexity of T cell motion. Instead, T cell movement is better described as a correlated random walk with a heavy-tailed distribution of step lengths. Using computer simulations, we identify three distinct factors that contribute to increasing T cell search efficiency: 1) a lognormal distribution of step lengths, 2) motion that is directionally persistent over short time scales, and 3) heterogeneity in movement patterns. Furthermore, we show that T cells move differently in specific frequently visited locations that we call “hotspots” within lymph nodes, suggesting that T cells change their movement in response to the lymph node environment. Our results show that like foraging animals, T cells adapt to environmental cues, suggesting that adaption is a fundamental feature of biological search.

## Author Summary

The immune system is responsible for clearing disease-causing infections, and T cells are an important immune cell type that helps eliminate viruses and bacteria. To become activated, T cells must encounter another type of immune cell called dendritic cells in the lymph node. T cell search for dendritic cells is similar to animal search for food. Here we precisely analyze how T cells move using search patterns originally developed to describe animals. We find that T cell motion is a complex combination of multiple strategies including moving in a persistent direction and using different step sizes. This allows T cells to balance the need to search both extensively throughout the lymph node and also to search some regions thoroughly for possible infection. Furthermore, we use a computer model to demonstrate that T cells are more likely to be found in specific locations in lymph nodes. We call these locations “hotspots”. We find that T cells in hotspots move differently, apparently searching more thoroughly, suggesting that T cells can adapt to their environment, similar to animals foraging for food. These results show that T cells share fundamental search strategies with foraging animals, exhibiting both persistence and adaptation.

## Introduction

Search has been extensively studied in biology, particularly in ecology, to understand how animals search for food, mates and prey. The pattern of movement by searching agents affects search efficiency in a variety of biological contexts [1–3]. Optimal foraging theory suggests that animals, including social animals such as ants and bees, have evolved strategies to individually or collectively maximize food intake in minimal time [4].

Similar to foraging animals, T cells of the immune system search for targets to mount an immune response. T cells are a critical immune effector, required to clear viral infections and to help B cells produce antibody. In order to initiate an effective immune response, naïve T cells must encounter and sample dendritic cells (DCs) bearing cognate antigen in lymph nodes (LNs). In the absence of infection, T cells continuously enter and exit LNs interacting with DCs. Upon infection, DCs present cognate antigen and provide stimulatory signals leading to T cell activation. T cell-DC interactions are required for naïve T cells to survive, activate and eventually clear infection as well as maintain immune memory [5–7].

T cell activation is promoted by repeated sampling of nearby DCs [8], while at the same time T cells explore the entire population of DCs for rare antigen indicative of infection. This presents T cells with an optimization problem in which T cells must balance *thoroughness* and *extent* of search. This requires that many T cells search across a broad *extent*, contacting many DCs quickly, a process similar to optimal foraging in animals. Simultaneously, T cell search is sometimes *thorough*, repeatedly sampling in a small area [8]. Both of these factors contribute to the overall rate at which T cells encounter DCs within LNs, which is a critical component of organismal fitness impacting the overall timing of the immune response.

Relatively little quantitative analysis has been done to describe how T cells move in LNs or how that movement affects the rate at which T cells encounter DCs. Initial studies to understand the type of T cell motion in LNs from pioneering two-photon imaging of naïve T cells suggested that T cells move using a simple diffusive random walk, analogous to Brownian motion of molecules [9,10]. Following these studies, computational modeling of T-DC interactions have often used simple diffusive random walks to represent T cell behavior [11,12]. However, subsequent studies have not precisely described T cell motion in LNs, so it is unclear whether diffusive random walks are appropriate models for T cell movement.

Optimal random search strategies have been extensively studied in ecology, and ecological models of movement may be useful for characterizing T cell motility and search efficiency. Brownian motion, Lévy walks, and correlated random walks (CRWs, also called persistent random walks), have been proposed as idealized biological search models [13], but careful quantitative analysis is required to understand how well search models characterize T cell motility and search efficiency [14]. Brownian motion is often referred to as a simple random walk and is characterized by movement with uniformly distributed turning angles and small fixed step sizes relative to the time resolution of observation [10,15–18]. Qualitative similarities between Brownian motion and the movement of microorganisms resulted in simple random motion being used as a dominant model of cell motion [19]. Brownian motion results in diffusive movement in which distance travelled is proportional to the square root of time. In two dimensions this results in a normal distribution of speeds, and in three dimensions it results in a Maxwell distribution of speeds [20].

Lévy walks exist between ballistic (or straight directional) motion at one extreme and Brownian motion at the other. In contrast to Brownian motion, the step lengths of Lévy searchers fit a power law distribution with most step lengths being small, but with a heavy-tail, that is, a decreasing probability of larger steps and a non-zero probability of steps of any length [2,13]. Lévy walks have been used to model animal movement, for example, in albatross, ant, aphid and human foraging, and more recently, T cells in the brain [2,21–24]. Both Brownian and Lévy walks assume that the direction of search at each step is drawn from a uniform distribution and is independent of previous steps (i.e. is isotropic and Markovian). CRWs on the other hand use fundamentally different mechanisms to model similar patterns of motion that tend to persist in direction over time. CRWs depend on the distribution of turning angles between successive steps leading to directional persistence. In search modelled by CRWs, the current direction of motion probabilistically influences future step directions [13]. On relatively short time scales, Lévy walks and CRW may be difficult to distinguish since they both produce superdiffusive motion [25], that is, displacement that increases faster than the square root of time. Compared to diffusive movement, superdiffusion increases search *extent* and decreases search *thoroughness*.

Despite the fact that many search strategies are well-characterized, there has been no systematic analysis of T cell motion in LNs. The lack of clarity in empirical studies has led to T cell motility being modelled using Brownian motion [18], Lévy walks [24], and correlated random walks (CRW) [8,26], or a combination of movement patterns [27]. Recently, Harris et al. showed that the movement of T cells in *Toxoplasma gondii* infected brain tissue fits a Lévy walk resulting in superdiffusion and efficient detection of protozoan targets [24]. It is not clear if Lévy movement has not previously been found in LN because such movement does not occur there, or simply because it had not been looked for. The lack of precise quantitative understanding of T cell motion in LNs leads to inconsistent models and limits our ability to determine how T cell motility affects the efficiency with which T cells encounter DCs.

In this study, we analyze T cell search behavior in LNs using two-photon microscopy. We begin our analysis with traditional statistical methods that describe the velocities, step lengths, displacement, and turning angles taken by naïve T cells searching for DCs. We then extend these analyses to more accurately and comprehensively describe motility patterns, including using maximum likelihood estimates (MLE) to fit experimental data. Our study statistically analyzes T cell search strategies in LNs, and uses multiple efficiency metrics that measure the spatial thoroughness and extent of T cell search. We then directly quantify the contribution of different types of motion to the efficiency of T cell search. Additionally, by comparing T cell movement to the patterns generated by null models of random motion, interesting non-random interactions between T cells and their environment become apparent, suggesting that T cells adapt movement in response to environmental cues. Our null models reveal hot spots that are visited more frequently than can be explained by chance. Our results suggest that even a precise characterization of T cell movement based on the assumption of random movement does not fully capture the complexity of T cell movement in the LN environment.

## Results

### Movement of naïve T cells in lymph nodes is superdiffusive, not Brownian

Two photon microscopy (2PM) has been used extensively to study the movement of T cells in intact lymph nodes [15,16,18,28,29]. We isolate bulk primary T cells from LNs of naïve C57Bl/6 animals, fluorescently label T cells with dyes, reintroduce labeled T cells into recipient mice, and then use 2PM to image labeled T cells in intact explanted LNs of recipients (see Materials and Methods for further details). We track cells for up to 10 minutes and include all motile cells in observation windows. We eliminate tracks with total track length shorter than 17μm or that show squared displacement less than 300μm^2^ (= 17μm x 17μm) as described previously by Letendre et al. [30]. The data analyzed here are from 5,891 individual T cell tracks from 41 fields from 12 experiments. We group those 41 fields into 7 datasets, each dataset containing fields imaged using frame rates within one second of each other. This allows us to combine data across fields when performing analyses, such as velocity autocorrelation, that depend on the frame rate.

We observe T cell velocities and motility coefficients largely in agreement with those previously published [9,16,30,31]. We calculate the diffusion coefficient using the unweighted average method [32,33]. T cells move with a mean speed with 95% confidence interval = 5.81 ±0.024 μm/min, median speed = 4.22 μm/min, motility coefficient, D = 19.2±0.534 μm^3^/min, calculated from a linear fit MSD of 5,185 tracks (out of 5,891 tracks filtered for *r*^*2*^ > 0.8). The motility coefficient is calculated using a linear model fit to the first 25% of each displacement curve and for positions not exceeding the 10 min track time.

**Fig 1:**
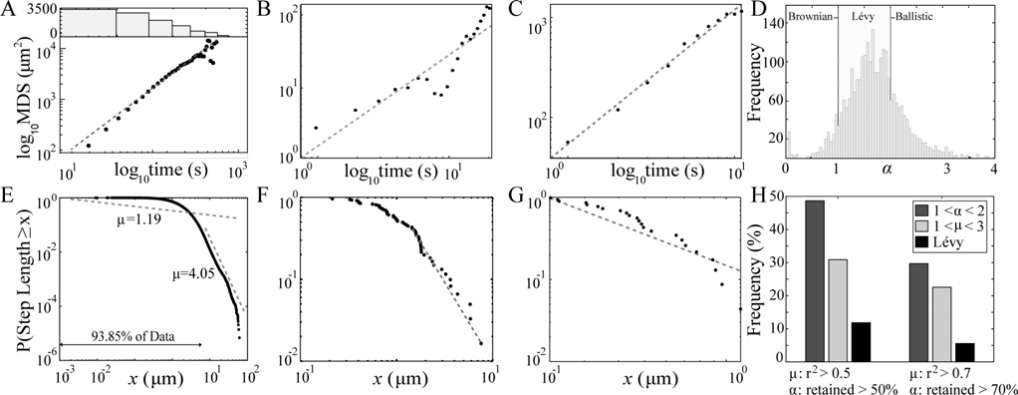
T cells move in lymph nodes with some features of a Lévy walk. Lévy walks are characterized by particular power law exponents of mean squared displacement (MSD) and step length distribution. (A, bottom) Observed T cell MSD vs. time. The dashed line is the linear regression with slope *α* = 1.41 indicating superdiffusion. (A, top) The number of data points in the MSD calculation. (B) Example displacements for a single T cell track with *r*^2^ = 0.52, and (C) with *r*^2^ = 0.93. (D) Histogram of *α* for individual tracks with *r*^2^ > 0.8 (see S2 Fig for other *r*^2^ thresholds) with labels indicating the range of values of *α* consistent with Brownian, Lévy and ballistic motion. (E) Empirical complementary cumulative distribution function (CCDF) of all 145,731 step lengths for all 5,077 cells. The *x*-axis is all possible distances less than the maximum observed, the *y*-axis is the probability that an observed step length exceeds a particular value of x. The dashed line (offset for clarity) with slope 4.05 is the best fit to the power law tail of the CCDF which includes only 6.15% of the steps [36]. The line with slope 1.19 is the best fit to all data. (F,G) Examples of step length distributions and MLE fits for tracks with 49% and 93% of the track in the tail. (H) Percentage of tracks in the Lévy region for *μ* and *α* power law exponents and their intersection. Data are included when the *r*^2^ > 0.5 for *α* and at least 50% (left histogram) or 70% (right histogram) of the track steps are retained in fitting the power law tail.

Displacement is commonly used as a first step to assess whether movement is consistent with a Lévy walk or Brownian motion (sample tracks in S1 Fig)[24,31]. We determine the displacement of individual T cells over time. Fig 1A shows the mean squared displacement (MSD) of one of the 7 datasets, as well example tracks with lower (Fig 1B) and higher (Fig 1C) *r*^*2*^ values. We then calculate the linear fit to the log-log-transformed data. Logarithmically transforming data before applying a linear regression is a common way to measure the exponent of a power-law relationship between dependent and independent variables [34]. Log-log-transformed Lévy walks produce displacement exponents, *α*, between 1 and 2 [35]. We calculate the distribution of *α* for all T cell tracks and find that 56% of T cells have a displacement exponent *α* falling in the expected window for a Lévy walk (Fig 1D). Only 28.3% of cell tracks are subdiffusive (*α* < 1), and the remaining tracks (15.6%) have a best-fit displacement exponent indicative of accelerating motion (*α* > 2). Because low *r*^2^ values of linear fits to log-log-transformed data may indicate that the data are not well-described by any displacement exponent, we repeat the analysis on data sets restricted to *r*^2^ values > 0.5, which discards 33% of all tracks, and *r*^2^ > 0.75, discarding 50% of all tracks (see S2 Fig for figures with different *r*^2^ filters). Increasing *r*^2^ filtering decreases the fraction of cells in the subdiffusive window, but the qualitative message remains the same: T cells demonstrate heterogeneous behavior, with some displacements consistent with subdiffusive, Brownian, ballistic and even accelerating motion, but the majority of T cells are superdiffusive but sub-ballistic. Fig 1D shows the histogram of *α* for tracks with an *r*^2^ > 0.8, other *r*^2^ thresholds are shown in S2 Fig, including all tracks with no filtering in S2A Fig.

### Naïve T cell movement in LNs is not consistent with a Lévy walk

While displacement analysis suggests many T cells are consistent with a Lévy walk, another defining feature of Lévy walks is that the inverse power law complementary cumulative distribution function (CCDF) for step lengths has an exponent, *μ*, between 1 and 3. Therefore, we analyzed T cell step lengths for the *μ* exponent. We define a *step* to be the resultant of a velocity subsequence in which each T cell velocity vector deviates by no more than 15° from the previous vector and a *step length* is the distance covered by a step. Fig 1E shows that a power law fit to the population of T cell step lengths is only valid if almost 94% of the data are excluded from the analysis (see Materials and Methods: Distribution fitting). The resulting best-fit *μ* exponent for the remaining 6% of the power law tail is 4.05 (Fig 1E). The curvilinearity, the poor fit, as well as the *μ* value all indicate that a Lévy walk is not a good description of T cell motility. On average 51% of data must be excluded in order to obtain a maximum likelihood estimated (MLE) power law fit (see Fig 1F and 1G for example tracks with low and high percentage of steps in the power law tail; see S3A and S3D Fig for histograms of *μ* using other goodness of fit (GoF) threshold values; and see S2 and S3 Figs for additional analysis.)

We determined the number of tracks that fit both 1< *μ* <3 and 1< *α* <2 parameters. Setting our GoF filtering criteria to require that at least 70% of the data per track is retained in calculating the exponent *μ* (Fig 1F), and the *r*^2^ statistic for the power law exponent *α* is at least 0.7, we find that only 5.5% of all T cell tracks fit both criteria for Lévy walk (Fig 1H). We note that the tracks excluded when filtering by *r*^2^ and those filtered by the percent of track in the power law tail both tend to be subdiffusive. For any filtering criteria the vast majority of T cell tracks are not Lévy walks (S3E Fig).

**Fig 2:**
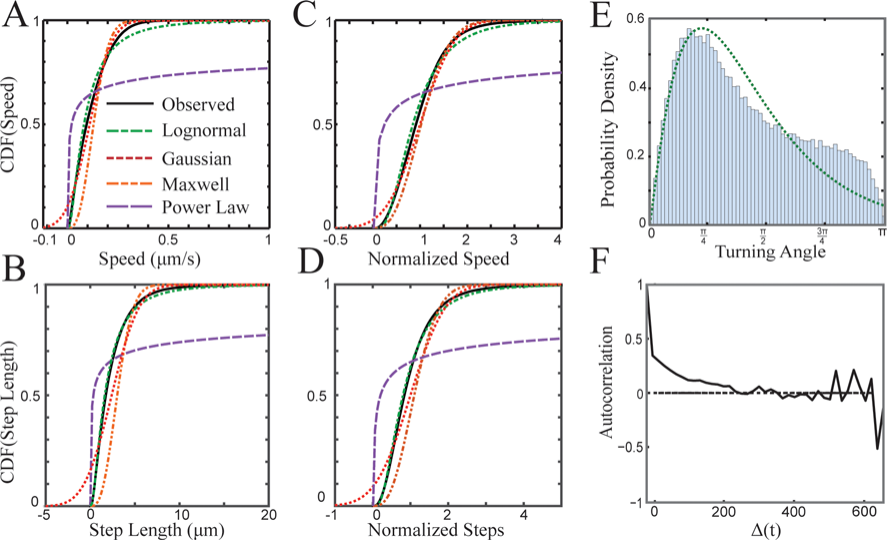
Distributions of T cell speed and step lengths with MLE fits. For (A) speed and (C) step length the lognormal function is the best fit (see Tables 1 and 2 for likelihood values and model parameters). Fits for normalized speed (B) and normalized step lengths (D) are divided by the mean speed or step length of the track from which they are drawn. (E) Histogram of all 149,592 observed turning angles. The green line is the maximum likelihood estimation of the gamma distribution used to model turning angles in the efficiency simulation. (F) Turning angle autocorrelation for 23,169 vectors from the 537 T cell tracks observed in one dataset. The correlation in movement direction decays until reaching zero at approximately 240 s.

To further analyze T cell motion, we quantify speeds (T cell displacement between consecutive frames multiplied by the frame rate) of all T cell tracks (Fig 2A and 2C) and find that in LNs T cell speeds range from 6.5×10^−4^ μm/s to 0.9 μm/s (Fig 2A). We fit experimentally derived speeds (Fig 2A) and step lengths (Fig 2B) to idealized probability distributions. We use parametric distributions because they are associated with well-known generative processes; for example, the Gaussian distribution is produced by the cumulative effect of additive processes, the lognormal distribution is often associated with multiplicative or branching processes [37], and the Maxwell distribution is a product of Brownian motion in three dimensions. We use likelihood measures to rank how well different distributions explain the observed data (Tables 1 and 2).

**Table 1:** MLE fits to step lengths and normalized step lengths (N = 145,731 steps). Negative log-likelihood measures the relative ability of candidate models to explain the observed data (For additional fits tested, see S1 and S2 Tables). The corrected Akaike information criterion (AICc) and Bayesian information criterion (BIC) (S2 Table) confirm that order of fit quality is not due to the number of model parameters. The most negative log likelihood and AICc scores are the best fits; in this case that is the smallest positive score for the lognormal distribution. The last column lists the distribution parameters that were selected by MLE. See S1 and S2 Tables for other distribution fits and goodness of fit statistics.

**Step lengths**				
Distribution	Negative log Likelihood×10^5^	AICc×10^5^	MLE Parameters	
**Lognormal**	**2.65**	**5.29**	**μ = 0.4818**	**σ = 0.9192**
Gaussian	3.36	6.72	μ = 2.3895	σ = 2.4229
Maxwell	4.02	8.04	a = 3.8497	
Power Law (Levy)	4.58	9.16	α = 1.1921	
**Normalized Step lengths (step length/track mean step length)**				
**Lognormal**	**1.20**	**2.40**	**μ = -0.2217**	**σ = 0.6896**
Gaussian	1.61	3.23	μ = 1	σ = 0.7324
Maxwell	1.69	3.37	a = 0.5117	
Power Law (Levy)	3.32	6.63	α = 1.2245	

**Table 2:** MLE fits to speeds and normalized speeds (N = 159,746). The lognormal distribution has the most negative log-likelihoods and AICc score and therefore is the best fit. The parameters selected by MLE are shown for each distribution. See S1 and S2 Tables for other distribution fits and goodness of fit statistics.

**Speeds**				
Distribution	Negative log Likelihood×10^5^	AICc×10^5^	MLE Parameters	
**Lognormal**	**-1.84**	**-3.68**	**μ = -2.5027**	**σ = 0.9329**
Gaussian	-1.61	-3.23	μ = 0.1161	σ = 0.0881
Maxwell	-1.12	-2.24	a = 0.0071	
Power Law (Levy)	0.122	0.245	μ = 1.2069	
Normalized Speeds (speed/track mean speed)				
**Lognormal**	**1.22**	**2.45**	**μ = -0.1669**	**σ = 0.6154**
Gaussian	1.37	2.74	μ = 1	σ = 0.5706
Maxwell	1.32	2.65	a = 0.4414	
Power Law (Levy)	3.58	7.16	μ = 1.2446	

The distribution of T cell step lengths and speeds is more consistent with a lognormal distribution than with Brownian motion (defined by a Gaussian or Maxwell distribution) or a Lévy walk (defined by a power law distribution of speeds [38]) as shown by the higher values in the MLE for power law fits in Tables 1 and 2. The variance of observed T cell speeds and lengths is high, and the distributions have a heavier tail (greater right skew) than both Gaussian and Maxwell distributions. The power law probability distribution over-represents both very small steps and very large steps compared to observed T cells. The lognormal distribution shows the best statistical fit for both speed and step lengths. The gamma distribution also fits the observed speeds very well (S1 and S2 Tables, S4 Fig). However since gamma and lognormal are often used to model the same phenomena, we present only lognormal here [39].

It is possible that the right skew in the speed distribution arises from the variance between track mean speeds rather than from speed variance within tracks [22]. To test for this possibility, we divide each speed drawn from within a cell track by the cell mean speed (called “normalized”) and ask whether the distribution becomes less heavy-tailed. We find that both normalized speed and step length distributions are still best fit by a lognormal distribution (compare Fig 2A with Fig 2C, 2B and 2D and the normalized vs. raw lengths and speeds in Tables 1 and 2), but the right skew is decreased. Our observations indicate that the heavy-tailed lognormal distribution is not simply due to distinct populations moving at different mean speeds, though heterogeneity in speed within the population is a factor.

Both Brownian motion and Lévy walks assume that the angle of each turn is drawn from a uniform random distribution. We analyze the turning angles of each T cell at each time step and find that T cell turning angles are not uniform, and that there is a bias toward turning angles of less than 90° (Fig 2E). The non-uniform distribution of turning angles suggests that T cells may move according to a CRW. We fit distributions to turning angles using MLE and find the gamma distribution to be the best fit, although it cannot capture all of the variation in the bi-modal distribution (Fig 2E green-dotted line). We then performed an autocorrelation analysis of directions over time to determine whether there is a dependency between the direction of T cells at one time step and the previous time steps (Fig 2F). We find that T cells show turning angle autocorrelation consistent with a CRW (indicated by positive values in Fig 2F). The correlation persists for approximately 4 minutes. Our cross-correlation analysis shows no drift in the observation fields (Materials and Methods: Equation 2).

### T cells balance search for unique individual targets and interactions with multiple targets

A key function of naïve T cell search within LNs is to find and interact with antigen bearing DCs. To determine whether different types of search can affect T cell interaction with DCs, we use an agent-based model, using biologically informed parameters, to assess the degree to which different modes of random search predict the observed pattern of T cell search efficiency (i.e., the number of DCs encountered per unit time). We reproduce features of T cell movement by creating search tracks using the best distribution fit to speeds (Table 2) and turning angles, limited by the total distance covered and time observed for empirical T cell tracks. We run simulations with DC targets placed with 3 different degrees of clustering: highly clustered (DC centers placed in 10μm radius spheres), moderately clustered (in 20μm radius spheres) to more evenly dispersed (in 40μm radius spheres) (S5 Fig). We confirm that these DC placements result in a range of clusteredness according to the Hopkins aggregation statistic that ranges from 0.44 for dispersed clusters (close to the 0.5 value expected for a uniform distribution) to 0.2 for compact clusters. We confirm that Brownian motion in our simulations results in diffusive movement (S6 Fig). We then compare efficiency of modelled search with observed T cell tracks from the experimental data across this range of DC cluster sizes.

We calculate efficiency of T cell search in two ways. First, we determine how many unique “DC” targets were encountered by each T cell in a specific period of time. Previous studies suggest that naïve T cells have no *a priori* information about the location of DCs in LNs [11,12]. Second, we determine how many total DC target encounters occur in the specified time. Total contacts count repeated contact with the same DC while unique contacts counts only one contact per DC. Total contacts are important for T cell activation and potentially survival while unique contacts are a measure of how long it may take T cells to find rare DCs presenting cognate antigen. The simulation addresses two questions: do statistical descriptions of T cell movement produce search efficiencies that are similar to those of observed T cells; and, how do the relative efficiencies of the idealized models compare to each other and experimentally observed T cells.

**Fig 3:**
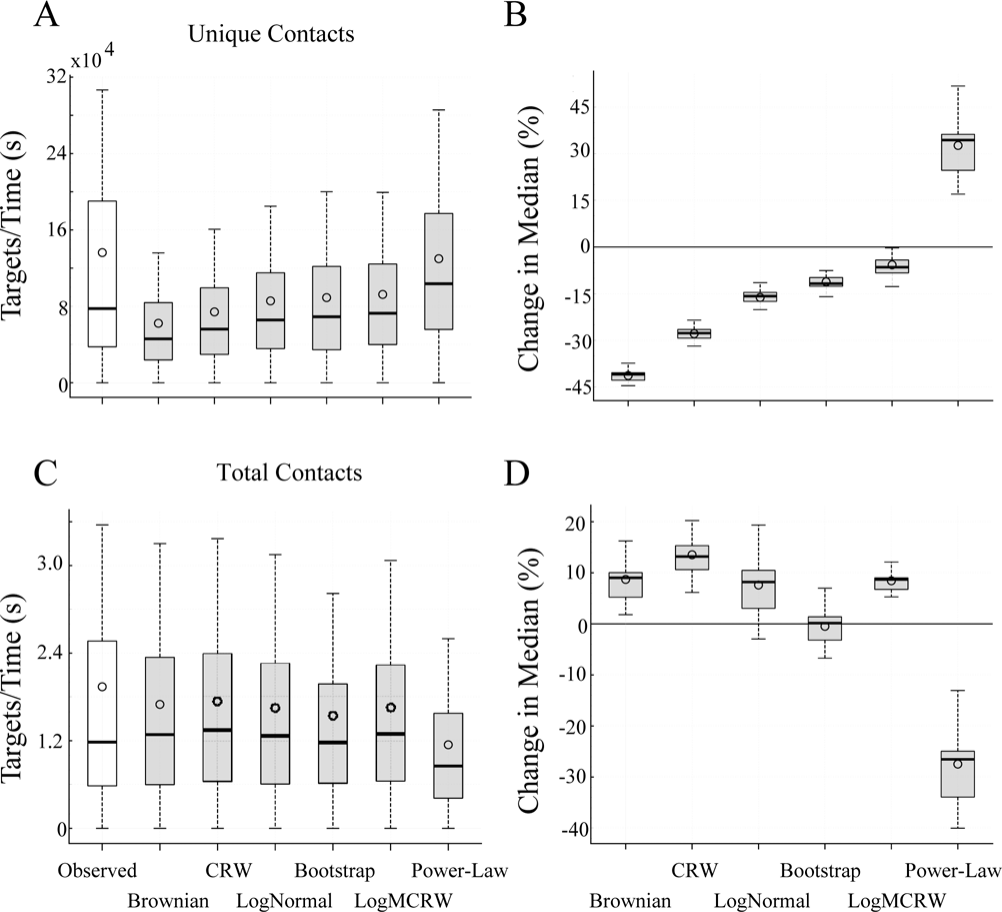
T cell search balances unique and total contacts with targets. Interquartile boxplots show search efficiency for DCs in 10 μm radius clusters. Panels (A) and (B) show *unique contact* efficiency; (C) and (D) show *total contact* efficiency. (A) and (C) show 1000 efficiency samples for each of the 41 fields. (B) and (D) compare the percent change in median search efficiency for each candidate search model relative to observed T cell search (indicated by the line at 0). See Tables 3 and 4 for other target distributions and significance values. Outliers are not shown for clarity.

Not surprisingly, the efficiency of observed T cells show a much wider range of variability compared with idealized models (Fig 3A), and we find clear differences in search efficiency between observed T cells and some idealized models. Brownian searchers are approximately 40% less efficient than observed T cells for unique DC contact (Fig 3A and 3B and Table 3). In contrast, the power law (Lévy) fit was 30% *more* efficient than observed T cell tracks, and as expected, more efficient than any other model for unique contacts with DCs. We also modelled a correlated random walk (CRW) as well as a CRW with a lognormal distribution of step lengths (a lognormal modulated CRW, LogMCRW). We show that the idealized search that most closely fits the observed efficiency of experimentally derived T cell search in LNs is the LogMCRW (Fig 3B), in keeping with CCDF fits (Fig 2). Efficiency is not dependent on placement of DC targets in the model: efficiency measures remain similar across multiple target distributions and degrees of clustering (Table 3). Thus, LogMCRW is not only the best description of the step length distribution, but also the best efficiency match for unique contact T cell search in LNs.

**Table 3:** Percent change of each idealized search strategy for unique contacts compared to the empirical search strategy across 3 different target distributions. Table entries are percent change in median search efficiency from observed ± 95% confidence interval. Two p-values are shown: the first indicates the significance of the change in *median* efficiency between the observed and idealized runs (N = 10 runs, each run consists of 4,100 samples, Fig 3B). The second p-value tests whether *all* raw efficiency values differ between observed and idealized runs (N = 41,000, Fig 3A). All p-values are calculated using the Mann-Whitney U test. The values in parentheses are the Hopkins aggregation statistic. All search strategies are statistically different from observations except LogMCRW in the most diffuse 40 μm DC clusters (in bold).

	Search Strategy					
Target Distribution	Brownian	CRW	Lognormal	Bootstrap	LogMCRW	Power Law
**10 μm (0.2)**	-41.88 ± 0.82	-28.11 ± 1.09	-15.91 ± 1.69	-12.98 ± 0.99	-7.35 ± 1.92	27.63 ± 5.44
	p < 10^−4^, 10^−4^	p < 10^−4^, 10^−4^	p < 10^−4^, 10^−4^	p < 10^−4^, 10^−4^	p < 10^−4^, 10^−4^	p < 10^−4^, 10^−4^
**20 μm (0.32)**	-39.828 ± 0.64	-25.87 ± 0.70	-13.39 ± 1.62	-9.926 ± 1.37	-3.98 ± 1.94	34.17 ± 8.49
	p < 10^−4^, 10^−4^	p < 10^−4^, 10^−4^	p < 10^−4^, 10^−4^	p < 10^−4^, 10^−4^	p < 10^−3^, 10^−4^	p < 10^−4^, 10^−4^
**40 μm (0.44)**	-41.88 ± 0.81	-22.75 ± 0.55	-9.621 ± 1.97	-4.798 ± 1.37	-0.218 ± 2.17	36.02 ± 6.41
	p < 10^−4^, 10^−4^	p < 10^−4^, 10^−4^	p < 10^−4^, 10^−4^	p < 10^−3^, 10^−3^	p = **0.85**, 10^−4^	p < 10^−4^, 10^−4^

Our simulation of unique target search also gives a quantitative estimate of the contribution of different types of T cell movement to search efficiency (Table 3). Correlation in angles of T cells increases the search efficiency by ~10% (from -42% for Brownian without correlation to -28% for CRW; -17% for lognormal to -7% for LogMCRW). The heavy-tailed step lengths contributed a 20% increase in efficiency (-42% Brownian to -17% lognormal). These results show that T cell motion is a complex mix of multiple motility parameters that contribute to overall T cell search efficiency.

In addition to unique antigen search, multiple DC contacts by T cells contribute to T cell activation and may also be required for survival [41–43]. Interestingly, we find that the efficiency of total contacts is reversed from that seen for unique contacts (compare Fig 3B and 3D, Table 4). Brownian searchers made the greatest number of total contacts, while power law (Lévy) searchers made the fewest total contacts (Fig 3D). Brownian searchers tend to resample the same locality and are therefore more thorough in their search at the cost of reduced search extent. In contrast, superdiffusive heavy-tailed searchers leave DC clusters more quickly and their total contact rate falls, increasing extent at the cost of thoroughness. Again, LogMCRW is closer to observed data than the other simulated patterns, and it successfully balances total contact rate with exploration of new DC clusters (Fig 3D).

**Table 4:** Percent change of each simulated search strategy for total contacts compared to the empirical search strategy across 3 different target distributions. Table entry format is identical to Table 3. These values correspond to Fig 3C and 3D. Brownian motion, bootstrap and LogMCRW are not significantly different from the observed distribution of efficiencies when targets are more clustered (in bold), but power law search underestimates the efficiency of search for total contacts.

	Search Strategy					
Target Distribution	Brownian	CRW	Lognormal	Bootstrap	LogMCRW	Power Law
10 μm (0.2)	8.7 ± 1.16	12.94 ± 1.34	7.24 ± 3.25	0.73 ± 2.59	8.4 ± 3.66	-28.66 ± 2.43
	p < 10^−4^, **0.29**	p < 10^−4^, 10^−3^	p < 0.01, 0.05	p = **0.63**, 10^−4^	p < 10^−3^, **0.73**	p < 10^−4^, 10^−4^
20 μm (0.32)	12.71 ± 1.52	15.67 ± 1.54	9.22 ± 2.72	2.29 ± 2.72	12.18 ± 2.64	-26.27 ± 4.14
	p < 10^−4^, **0.87**	p < 10^−4^, 10^−4^	p < 0.05, 0.05	p = **0.19**, 10^−4^	p < 10^−4^, **0.8**	p < 10^−4^, 10^−4^
40 μm (0.44)	17.71 ± 1.86	20.89 ± 1.58	13.07 ± 3.24	4.52 ± 2.51	16.31 ± 2.69	-24.08 ± 4.4
	p < 10^−4^, 10^−4^	p < 10^−4^, 10^−4^	p < 10^−4^, 10^−4^	p < 0.05, 10^−4^	p < 10^−4^, 10^−4^	p < 10^−4^, 10^−4^

We also performed a statistical bootstrap analysis in which search tracks were generated by sampling uniformly from all observed track speeds and turning angles [41]. While the efficiency of total contacts for bootstrap tracks is statistically indistinguishable from observed T cells, bootstrap tracks are 12% less efficient than observed cells in unique contacts (Fig 3B and Table 3). Thus, individual T cell tracks confer efficiency for unique DC target search that is lost when the steps within a track are randomized, suggesting that there is underlying heterogeneity in T cell tracks that increases T cell search efficiency.

### Naïve T cells show heterogeneity in movement patterns

**Fig 4:**
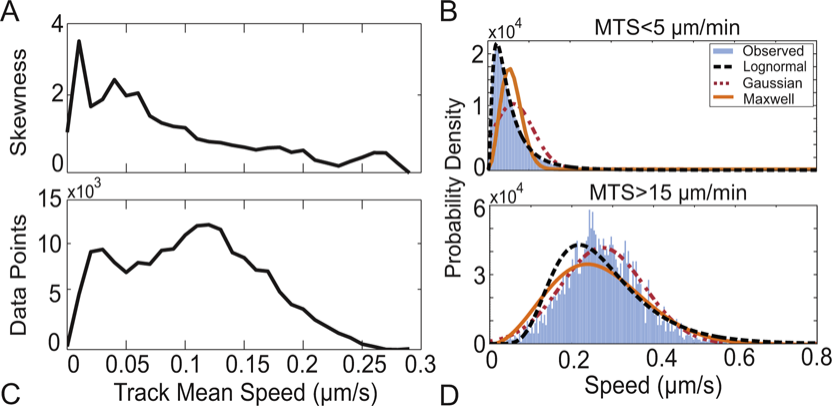
T cells moving at different speeds show different movement patterns. (A) Skew of step length distribution as a function of track mean speed and (C) the number of data points as a function of track mean speed. Tracks with mean track speeds (MTS) less than 5μm/min (B) and greater than 15μm/min (D) were selected to illustrate different MLE model fits for fast and slow tracks (for fits see Table 5).

To assess potential variation in T cell motility, we analyzed differences in speeds across individual T cell tracks. We find that the distribution of speeds is highly skewed for cells with lower mean speeds, but there is less skew for cells with high mean speeds (Fig 4A). The fastest cells (mean speeds >15 μm/min, Fig 4D) produce more symmetric speed distributions as demonstrated by the low skew and kurtosis. Also, distribution fitting of speeds shows that the speeds are now best fit by Gaussian and Maxwell distributions (Table 5). In contrast, slow cells (mean speed <5 μm/min, Fig 4C) have a heavier tailed distribution of speeds as shown by skew and kurtosis with lognormal remaining the best fit (Table 5). This is not due to the number of data points available at high speeds, as skew decreased even at the speeds with the highest number of data points (Fig 4B). However, “slow” and “fast” are not discrete populations, as a mixed Gaussian cluster analysis shows no evidence of discrete populations defined by mean speed and variance (S7 Fig). These results suggest that T cells exhibit a continuum of movement patterns within LNs, leading to different types of searches: slow moving cells show a heavy-tailed distribution while faster moving cells are more Brownian.

**Table 5:** Best fit likelihood and MLE estimated parameters for the fastest and slowest cells. The Gaussian distribution better fits tracks with mean speed > 15 μm/min while lognormal better fits tracks with mean speed < 5 μm/min. The step speed distribution for fast tracks has a shorter and lighter tail than the sample of tracks with slower mean speeds. Best negative loglikelihood scores are in bold. For tracks with mean speed of < 5 um/min, skew is 2.37 and kurtosis 13.3; for tracks with mean speed > 15 um/min, skew is 0.52 and kurtosis 3.98.

	Mean Speed < 5 μm/min			Mean Speed > 15 μm/min		
Distribution	-log Likelihood (×10^5^)	MLE Parameters		-log Likelihood (×10^3^)	MLE Parameters	
**Lognormal**	**-1.09**	**-3.35**	**0.826**	-2.60	-1.35	0.387
**Gaussian**	-0.918	0.0482	0.0407	**-2.73**	**0.277**	**0.0958**
Maxwell	-0.789	0.0013		-2.66	0.0286	
Power Law	-0.492	1.25		2.018	1.35	

### “Hotspots” in the LN environment show differing patterns of T cell motion

The variation in movement shown in Fig 4 suggests that T cells may alter their search pattern in response to environmental cues. Our previous work shows that altering movement in response to environmental cues can enhance search efficiency [44,45]. Extending our previous work in [46], we analyze T cells in LN to identify whether T cells movement changes within local microenvironments of the LN. To do this, we identify whether there are locations in the LN that are visited by T cells more frequently than predicted by a null model. We analyzed each observation field separately; each field was discretized into cubes of 20μm per side, which is approximately twice the diameter of a naïve T cell. We used the LogMCRW simulation we described earlier as a null model (for details of null model see Materials and Methods, S11 Fig). We identified spots that were visited by T cells in the simulation null model and then identified spots that were visited by T cells from actual experimental data. We found that experimental T cells visited certain spots at significantly higher frequency than the null model (see S11 Fig [47]). Spots that were visited at a frequency 2σ higher than the null model were called “hotspots” (examples shown in S12 Fig). Hotspots were observed in 37 of the 41 observation fields. The null model results in only 2.73% of visited locations being hotspots (as expected given that we identify hotspots as those visited 2 standard deviations above the mean, S11 Fig); in contrast, in empirical observations, 10.51% of locations from observed experimental data are hotspots. We also find that our null simulation predicts 32% tracks will visit hotspots but our observed tracks show that 51% of observed tracks visit hotspots. These data all support the hypothesis that hotspots exist in empirical observations.

**Fig 5:**
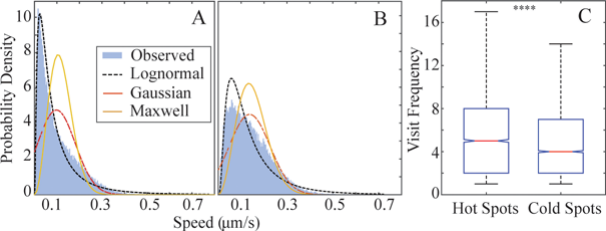
T cells visiting hotspots show a different distribution of speeds than T cells that do not visit hotspots. Cold tracks (A) have a speed distribution that is more peaked at low speeds with a more skewed, heavy-tailed distribution compared to hot tracks (B). For fits, see Table 6. (C) Visit frequency, or number of observations of hot tracks in hot vs. cold spots. Hot tracks were observed to visit hotspots more than cold spots. The graph shows the distribution of average number of visits by hot tracks to hotspots versus cold spots. Interquartile box plot of the distribution with the red line indicating the median number of visits. Outliers are not shown. **** indicates p<<10^−3^ using Mann-Whitney U test.

We define *hot tracks* to be T cell tracks that intersect with hotspots and *cold tracks* to be those that do not. Hot tracks have median speeds that are significantly higher than cold tracks with hot track speed at 7.27 μm/min and cold tracks at 4.25 μm/min (median speed is 37.4% greater for hot tracks than for cold, p-values << 10^−3^ Mann-Whitney U test). We also find that the step length distributions of hot tracks have a significantly lower skew and kurtosis compared to cold tracks (Table 6), indicative of more Gaussian distributions in hot tracks. Furthermore, though the step lengths of hot tracks and cold tracks are both best fit by lognormal PDFs, the Gaussian and Maxwell distributions are nearly as good for hot tracks (Fig 5A and 5B and Table 6). These results show that T cells that visit hotspots exhibit different, and more Brownian movement, suggesting that they search more thoroughly than T cells that do not visit hotspots.

**Table 6:** Hot and cold track step lengths show different MLE distribution fits. Hot tracks tend to be faster than cold tracks and more Brownian in their movement pattern. The high kurtosis and skew is due to a long tail in the distribution of step lengths belonging to tracks that do not visit hotspots. For hot tracks, skew is 1.45 and kurtosis 6.74; for cold tracks skew is 11.12 and kurtosis 136.

	Hot Tracks			Cold Tracks		
Distribution	-log Likelihood (×10^5^)	MLE Parameters		-log Likelihood (×10^5^)	MLE Parameters	
Lognormal	1.29	0.671	0.752	1.85	0.819	**0.583**
Gaussian	1.405	2.504	1.72	4.22	4.53	**22.3**
Maxwell	1.48	3.077		8.24	171	
Power Law	2.96			2.65	1.21	

The presence of hotspots suggests that a microenvironment within the LN might modify T cell behavior. To show T cell adaptation within LNs, we ask whether hot tracks (T cells that have visited hotspots) behave differently in hotspots versus other locations within the LN (cold spots). We find that T cells from hot tracks spend more time in hotspots than in other locations (cold spots), with T cells spending a median of 5.36 time steps in hotspots compared to 4.5 in cold spots (p-values << 10^−3^ Mann-Whitney U test, Fig 5C). T cells that visit hotspots are found in those hotspots between 13.3% and 23.2% (95% confidence interval) more often than they are in other LN locations, i.e. cold spots. Thus, hotspots are visited by more T cells than can be explained by chance, the T cells that visit those hotspots move differently than those that don’t, and T cells spend more time in hotspots than in other locations; all suggesting that T cell movement changes in response to the LN environment.

## Discussion

T cell activation depends on interactions between T cells and antigen-bearing DCs in secondary lymphoid organs including LNs [9,17]. In this study, we quantify the movement of T cells within LNs, and how efficiently they encounter DC targets (in terms of both unique and total contacts). We use quantitative analysis and computer simulations to show that a search strategy that employs both correlations in successive turning angles and a lognormal distribution of speeds is most representative of observed T cell motion, which we call a LogMCRW. However, T cell motion does not perfectly fit any simple parametric model, and different types of motility are observed depending on where the T cell is and how fast the T cell moves.

Accurate characterization of T cell movement is important because motility determines the timing of other immune processes downstream of T cell activation. Several groups have published models of how T cells interact with DCs in LNs. Mirsky at al. [48] provide a review. Recent data also suggests that motility can affect both T cell recirculation [49] and T cell dwell time leading to activation especially when detecting rare antigen [8,41]. Different studies employ different models of T cell motion due to the lack of precise understanding of how T cells move. For example, some models assumed Brownian movement while another assumed a CRW with a Gaussian distribution of steps and speeds, and yet another uses tracks bootstrapped from empirical data [18,26,40,41]. Our results show that the LogMCRW pattern of motion not only fits the experimental data, but also most faithfully reproduces the modelled search efficiency of observed T cell movement.

We use an agent-based model to compare empirical T cell movement to idealized simulations. These simulations demonstrate that simulated Lévy walks overestimate real T cell search efficiency (for unique DC contacts) while the Brownian walk, CRW, and bootstrap tracks underestimate it. The reverse is true for total contacts. A lognormal distribution of steps combined with correlation among steps (LogMCRW) best represents empirical T cell search efficiency for total and unique contacts.

We identify and quantify three mechanisms that increase T cell search efficiency for unique targets: 1) heavy-tailed step lengths (comparing lognormal versus Brownian search accounts for 20%); 2) directional correlation (comparing lognormal vs. LogMCRW accounts for 10%); and heterogeneity among T cells (comparing bootstrap to observed accounts for 10%) (Fig 4 and Table 3). Thus, computational models allow us to quantify the contribution of a variety of factors to T cell search efficiency.

In our study, we thoroughly analyze the motility of naïve T cells in LNs in the absence of antigenic stimulation. Our results largely agree with a recent study by Banigan et al. also showing persistent directional movement for 3–4 minutes by naïve T cells [27]. T cells have previously been shown to move in “streams”, which may correspond to the persistence in movement. Persistence may also reflect cells following a path of least resistance or intrinsic regulation of cell movement, for example, the time required to form a leading edge.

In contrast to Banigan, we find a lognormal distribution of T cell steps and show that the heavy tailed distribution of step lengths is important for search efficiency. Banigan et al. also suggested that modeling T cell movement using 2 subpopulations may be a more faithful reproduction of T cell movement in LN [27]. Our data does not support the existence of 2 subpopulations of T cells. Rather, we find that there may be subregions (hotspots) within the LN that leads to differences in T cell search behavior. T cell motion near hotspots is less directionally persistent and more Brownian (Fig 5). These results demonstrate that T cells react to their environment, and more specifically, they suggest that T cells that visit hotspots stay longer and thus search more thoroughly at those hotspots.

The identity of hotspots remains to be determined. It is possible that hotspots are locations of DCs or high endothelial venules from which T cells enter the LN. T cells that search areas with DCs more thoroughly may have more repeated contacts with the same DC as well as contacts with more DCs within the same area, enhancing the potential for productive T cell interaction with DCs presenting cognate antigen. One potential mechanism for hotspots is chemokine production by DCs, although there is no direct experimental evidence for this. Another possibility is that hotspots may reflect an underlying structure such as the fibroblastic reticular cells that may form a network that guides T cell movement [50]. However, the distribution of our hotspots does not obviously reflect any network structure. Others have tested the potential role of a network on T cell search efficiency [11,51] and found that the presence of a network has little impact on T cell search efficiency.

Upon activation by cognate antigen, T cell motility within the LN changes, T cells slow down over a period of several hours and begin to form long lived interactions with DCs, essentially ending the “search” phase [16,17]. Effector T cells then exit the LN and enter peripheral sites of inflammation. Effector T cell motion in the brains of *Toxoplasma gondii* infected animals was shown to be a generalized Lévy walk based on displacement analysis [24]. This differs from our findings that T cells in LNs do not fit a Lévy walk. The difference between our findings and those of Harris et al. may result from intrinsic differences between naïve and effector states. Another possibility is that differences between the tissues that the T cell resides in, for example, the LN for naïve T cells or the brain for effector T cells, contain structural and chemical variability leading to different motility.

As expected, our simulation shows that Lévy searchers are efficient at finding rare targets, but Brownian motion is more efficient when measuring total contacts. These results show that biological context may be important for T cell search efficiency: in the search for rare and unique antigens, the heavy-tailed search is more efficient. However, in situations where high numbers of DC contacts may be important for T cell activation and potentially survival, Brownian motion has an advantage. The observed T cell motion appears to combine the best properties of each, utilizing multiple modes of motility to achieve efficiency in different contexts.

Previous studies have used modeling to reproduce experimental results, and we use this approach to show that the LogMCRW statistical model captures immunologically important properties of T cell search. Similar to empirically observed T cell movement, combining multiple features of random search in the LogMCRW balances search over a wide spatial extent to find unique targets, with thorough search that allows repeated contacts within a cluster. In addition, we extend our use of modeling to identify novel features of the biology underlying T cell movement in LNs. Because the LogMCRW is a good estimate of search efficiency, it also provides a useful null model with which observed T cell motion can be compared, revealing that T cells move differently in different locations in the LN. Thus the statistical model and search efficiency simulations not only characterize cell movement and provide estimates of search efficiency, they can also be used to reveal the complexity of T cell motility.

Indeed, comparison to our null model reveals non-random T cell movement which may indicate change in response to some feature of the LN. We find that T cells respond differently to specific microenvironments within the lymph nodes, which we call hotspots. The presence of hotspots suggest that, like foraging animals, T cells may respond to features of their environment in order to guide their search [52,53].

Prior work has characterized the movement of foraging animals using both CRW and Lévy walks. Lévy walks in particular have been suggested as optimal to maximize foraging rate [2,16]. Our work suggests that in order to balance maximizing repeated (total) contacts with maximizing new (unique) contacts, the LogMCRW may be more effective. More generally, walks with heavy tailed step length distributions and correlation among turning angles may be most effective at balancing the thoroughness and extent of search. In foraging animals as well as searching T cells, natural selection may opt for movement that is effective in a variety of circumstances, even if that movement is difficult to describe analytically.

T cells provide a unique window into biological search strategies because so many searchers can be visualized rapidly in relatively intact natural conditions. Such movement patterns can be included in agent-based models, even if they are not easy to present in closed form equations. Our data suggests that the LogMCRW strategy might be a better approach than either Brownian or Lévy walk in situations that need to balance repeated contacts with already-found targets and discovery of new items. Additionally, T search for patchily distributed DCs [16] in the LN may demonstrate response to cues, similar to other collective foragers such as ants collecting patchily distributed resources in natural habitats [54].

In contrast to previous assumptions about simple random motion, our analysis shows that T cell movement in lymph nodes is complex, and involves correlation, variation in step lengths, and heterogeneity in response to local environments. The deviation from idealized models reflects the immunological need to balance the spatial extent and local thoroughness of search. The complex movements of T cells in LN provide a window into biological search strategies and how natural selection may balance multiple objectives in a variety of biological contexts.

## Materials and Methods

### Ethics statement

The protocol was approved by the IACUC at the University of New Mexico (protocol # 10–100487). The breeding and maintenance of mice used in this research conform to the principles outlined by the Animal Welfare Act of the National Institutes of Health. All efforts were made to minimize suffering with use of ketamine and xylazine when appropriate. Euthanasia was performed by isofluorane overdose.

### Mice

C57BL/6 mice were from Jackson Laboratories (Bar Harbor, ME). All mice were bred and/or maintained in a specific pathogen-free condition in barrier facilities (Albuquerque, NM) and conform to the principles outlined by the Animal Welfare Act and the National Institutes of Health guidelines.

### T cell observations using two-photon microscopy

Lymph nodes were prepared according to the protocol described previously [30,55–57]. T cells were purified by nylon wool or by negative selection using the pan-T cell kit (Miltenyi Biotec) as previously described by Cannon et al. [28] and purified T cells labeled with either 1μM CFSE (Invitrogen) or 5 μM CMTMR (Invitrogen, Carlsbad, CA). 5 to 10×10^6^ labeled T cells were injected I.V. into recipient mice and inguinal lymph nodes were removed 15–18 hours later and imaged using two photon-imaging.

Imaging experiments were performed using either a workstation with a Bio-Rad Radiance 2000 scanner mounted on an Olympus upright microscope with a chamber at 37°C or a 2-photon microscope in the Fluorescence Microscopy Facility in the UNM Cancer Center with a mode locked Ti:Sapphire infrared laser (Coherent Ultra II; tunable from 680–1080 nm; avg. power 3.5 W) for multiphoton fluorescence excitation on a Zeiss Axiovert 200 stand. For the Bio-Rad 2P, explanted lymph nodes were placed on a glass coverslip in the chamber. The sample is perfused with a 37°C solution of DMEM (phenol red free, Gibco) bubbled with 95% O_2_ and 5% CO_2_. T cell motility within a lymph node was monitored in the T cell area at a minimum of 50–70μm below the surface of the node. For the Zeiss 2P, the microscope stand is a Zeiss Axiovert 200 with motorized XY stage and IR-corrected long working distance objectives (25X:multi-immersion and 40X:water immersion) and image acquisition via a Zeiss LSM510 scanhead. Ex-vivo tissue and organs are maintained during microscopic observation in a stage microincubator system (LCI-Live Cell Imaging) equipped with heating, humidity, CO_2_ atmosphere and perfusion. Explanted lymph nodes were placed on a glass coverslip in the chamber. The sample is perfused with a 37°C solution of DMEM (phenol red free, Gibco) bubbled with 95% O_2_ and 5% CO_2_.

For 4D analysis of T cell motility, multiple stacks in the z-axis (z step = 3 μm) were acquired every 15–20 s (depending on the number of z stacks acquired) for 15–40 min, with an overall field thickness of 40–60 μm. Cell motility was analyzed with Imaris software (version 6; Bitplane). Tracks that lasted fewer than 3 time steps (duration filter in Imaris) were not taken into account in the analysis. Length filter (threshold of 17 μm = 3 times the diameter of the cell) Displacement^2^ filter (threshold of 300 μm^2^ = 17 μm X 17 μm) were also used to discard tracks of non-motile cells. Videos were made by projecting the 4D information along the z-axis in a single plane.

The observation area covers approximately two thirds of the T cell zone of the lymph node. Cell motility was analyzed with Imaris 6.0 (Bitplane AG, Zurich, Switzerland). The point sequences generated by Imaris were used to create position vectors joining adjacent cell locations (sample tracks S1 Fig). The Euclidean norm for each vector was calculated and divided by the time resolution to produce speeds.

### Distribution fitting

Following Fisher [58] we use maximum likelihood estimation (MLE) to parameterize candidate PDFs. We fit probability model parameters using cumulative distribution functions (CDF), rather than by binning data which has been shown to bias conclusions about random walk distributions [59]. We define a *step* as a vector of T cell motion that does not deviate beyond 15° from the original direction (see S8 Fig for analysis of threshold dependency).

Five PDF models (lognormal, Maxwell, Gaussian, exponential, and power law) for step length and speed were selected for analysis based on a combination of their negative log-likelihood scores, their importance in other biological processes, and their previous use in modeling T cell movement. Our selection of the relative goodness of fit (GoF) of each candidate PDF to empirical data was evaluated using likelihood functions, Anderson-Darling (AD), Bayesian information criterion (BIC), corrected Akaiki Information Criterion (AICc), and the Kolmogorov-Smirnov (KS) test.

Following Clauset, Shalizi, and Newman [36], we fit power laws using MLE and with the power law PDF: $P(x)=\frac{\mu -1}{x_{\text{min}}}{\left(\frac{x}{x_{\text{min}}}\right)}^{\mu }$ where *x*_min_ is the smallest observed value, *P*(*x*) is the probability of *x* occurring, and *μ* is the estimated parameter. We used the *x*_min_ value with the best KS score of all possible choices as an estimator of the beginning of a power law tail. The percentage of positions in a track in the power law tail gives us a measure of the quality of the power law fit. Using this measure we show that a power law fit to the population of observed steps excludes 94% of the data (Fig 1F and 1H).

### Autocorrelation and cross-correlations

Velocity autocorrelations were calculated following [60] and [61]. The autocorrelation function is the ensemble mean for the *n-1* possible delay times given the *n* vectors defining a T cell track. The result is a measure of how much T cell direction depends on previous directions as a function of time delay. Our use of autocorrelation is distinct from the analysis of periodic velocity vector magnitudes by Beltman et al. [40], but the similar to that done in Banigan et al. [27].

Letting *v*(*p*_*k*_(*t*)) be the unit velocity vector at time *t* belonging to the *k*^*th*^ path, we defined the cross-correlation function, C_cross_, to be: C_cross_(*p*) = ⟨*v*(*p*_*k*_(*t*)) ∙ *v*(*p*_*m*_(*t*))⟩, ∀*k*, *m* where *p*_*k*_ and *p*_*m*_ are T cells paths. This measures the step angle dependence between T cell paths at the same moment in time, that is, a measure of drift due to global effects on the observation field.

### Mean squared displacement

Mean squared displacement (MSD) coefficients, commonly called the *α* exponent [1,13,21], were calculated using least-squares polynomial fit by numerically solving the associated Vandermonde matrix [62] and fit quality assessed with the *r*^2^ measure. Parametric and linear fits were also made to mean displacement. In Fig 1A we present only the first 10 minutes of observation (as was done in [16,63,64]) at which point the curve reaches its first stationary inflection which in [61] is indicative of unconstrained motion and therefore appropriate for determining *α*. In addition, in this study few tracks persist beyond 10 minutes and so the MSD signal also becomes dominated by noise (Fig 1A top).

### Heterogeneity

We tested for heterogeneity by comparing track speed skew (Fig 4) and AIC evidence ratios as a function of mean speed. The sample skew of the distribution of speeds was calculated using the method of moments applied to a mean speed sliding window of width 0.125 μm/s progressing in 0.1 μm/s increments.

### Search efficiency simulation

The simulation to test T-DC interaction efficiency was implemented as a continuous (floating-point) 3D model written in C++. Boost libraries [65] were used to generate variates drawn from model PDFs. Because the clustering and density of targets can influence which movement types are most efficient, we replicated the estimated density of DCs and varied the degree of clustering in our simulations.

We use LN DC density of 2–5% as determined in [43] to calculate a target DC density of 3.17×10^−5^ targets/μm^3^. Our observed fields have an average volume of 6.3×10^6^ μm^3^. We scale the number of targets as a function of field volume in order to maintain the same target density between simulation fields. DCs were clustered into groups of 10 and were uniformly distributed within spheres defining a cluster. By varying the sphere radius, we controlled the degree of clustering from uniform to highly clustered. A 3D version of the Hopkins statistic [66] was used to measure the resulting non-uniformity of target placement (Tables 3 and 4). In the Hopkins statistic scores range from 0 to 0.5 where 0 is highly clustered and 0.5 indicates no clustering (S5 Fig).

T cell tracks were observed and recorded as 3D coordinate sequences within a bounding box defined by the visible section of the *ex vivo* lymph node. Idealized models (Brownian, CRW, Power Law, etc.) of search were parameterized by the speeds and turning angles estimated from observation (see Distribution fitting). Searchers in the idealized model start at the same initial positions as the observed T cells, and exist in a volume equal to the observed field volume. Candidate search patterns were generated for each of the 41 observation fields.

Our efficiency measure is the number of targets found divided by the sum of the time used by searchers. Since we modelled walks rather than flights (i.e. speeds are finite) the sum of D(*k*) for all simulated tracks *k* was limited to the total distance travelled by observed T cells. Therefore the average velocity of the population of searchers is kept within the observed range. Based on an assumed radii of 5 μm for DCs and T cells, targets were marked as discovered if a searcher track passed within 10 μm of a target point. We define two versions of the efficiency measure, one that increments its output value only when a target was not previously detected by that searcher, and another that increments for all targets found. These two versions allow us to record unique contacts and total contacts (Fig 3).

The simulation measures the target encounter rate and determines, using the Mann-Whiney test, whether the candidate search models’ search efficiency is significantly different from that observed in T cells. We use the Mann-Whitney test because the observed and simulated distribution of efficiencies is non-Gaussian. Simulations were replicated 100 times per field, producing 4,100 efficiency data points for each search model. The entire process was repeated 10 times in order to generate confidence intervals for the simulation; in all this results in 41,000 efficiency samples.

### Identifying hotspots and hot tracks

In order to test whether the environment within LNs influences T cell movement we extend an analysis begun in [46]. Fields were discretized into 8000 μm^3^ cubes (the length of a cube is 20 μm, approximately twice the diameter of a T cell). We use the LogMCRW simulation as a null model and record the number of times a location is visited by unique T cells in simulation (repeated 10 times). We use a 2σ (two-standard deviation) threshold for determining which locations are visited more frequently in the observed fields than expected and call these hotspots. This is repeated for each of the 41 individual observational fields. All other visited locations are called cold spots. A comparison of the number of hotspots in simulation and in the observed data gives an indication of how much behavior is not captured by the simulation.

We define hot tracks to be T cell tracks that visit hotspots and cold tracks to be T cell tracks that do not. We also examine the number of visits by hot tracks to cold spots and hotspots. We also examine the distribution of step lengths and speeds for hot and cold tracks.

For additional information on methods, see supplementary materials and methods (S1 Text).

## Supporting Information

S1 Text Supplemental Methods. (DOCX)

S1 Fig Example of individual T cell tracks. (TIF)

S2 Fig Histogram of mean squared displacement exponents with varying *r*^2^ filters. As the linear regression slopes are filtered by the *r*^2^ statistic, the histogram narrows but maintains its mean value. (A) *r*^2^ > 0, 3.5% of tracks filtered, (B) *r*^2^ > 0.25, 21% filtered, (C) *r*^2^ > 0.5, 33%, (D) *r*^2^ > 0.75, 50%, and *r*^2^ > 0.9, 69% of tracks filtered out. (E) *r*^2^ > 0.8 with regions of interest marked.
(TIF)

S3 Fig **Histogram of power law exponents fit to the CCDF of step length for tracks with varying percentages of their steps in the power law tail: (A) all tracks, (B) tracks with at least 50%, (C) 70%, and (D) 90% of steps in the power law tail.** An increasing fraction of steps in the tail results in *μ* values being more likely to be between 1 and 3 but as a total fraction of all tracks those well fit by a power law falls rapidly, for (A) 35%, (C) 31%, (D) 24%, and (E) 7% of total tracks are represented. (E) Fraction of Tracks with Lévy characteristics. Power law exponents, *μ*, for step length and *α*, for displacement. Tracks are grouped by fit quality (GoF). Retained percentage refers to the amount of data discarded in order to obtain a power law fit (see methods for *μ* fitting). Displacement *α*, values are filtered by *r*^2^.
(TIF)

S4 Fig Weibull probability plot. The gamma probability distribution has comparable negative log-likelihood scores to the lognormal distribution (speeds shown here). The lognormal model overestimates the probability of high speeds at the tail of the distribution while the gamma distribution over estimates the probability of very low speeds.
(TIF)

S5 Fig Sample DC target cluster distributions in simulation. Panel A: 20 μm radius clusters with Hopkins index = 0.2. Panel B: 20 μm radius clusters with Hopkins index = 0.32. Panel C: 40 μm radius clusters with Hopkins index = 0.44.
(TIF)

S6 Fig Mean squared displacement for simulated search models. Numbers in color indicate the slope of the mean-squared linear fit to the log-log transformed displacement curve. As expected, Brownian motion has a slope close to one, as does the lognormal step distribution model. All other models produce superdiffusive motion.
(TIF)

S7 Fig We found no evidence of distinct subpopulations defined by variance and mean speed. An expectation maximization Gaussian mixture model finds that clustering tracks according to track speed and track variance results in a single grouping. The color bar and contour map indicate the height of the best-fit Gaussian model. Increasing the number of Gaussians to fit incrementally up to 16 does not reveal any natural clusters. This figure supports the skew plot Fig 4C. Example field (1 of 41).
(TIF)

S8 Fig The dependency between the angle used to calculate steps from T cell positions and the number of steps resulting. For example at threshold of 180° all steps in each track are combined and the resulting number of steps in the population is small. The influence of the angle threshold on the number of combined positions is smooth. No natural choice of threshold angle is apparent.
(TIF)

S9 Fig As the number of data points in tracks lasting more than 10 minutes drops, MSD becomes dominated by noise. As a result we perform linear regression only on the first 10 minutes of each track (green line). (1 of 7 datasets).
(TIF)

S10 Fig Visualization of search tracks. Dark green targets are undiscovered. Targets become cyan if they are within the search volume of a T cell track (detected). In this example targets are grouped into clusters of 10 with radius 10 μm. Each T cell track is assigned a random color to help distinguish them from one another. Example field (1 of 41).
(TIF)

S11 Fig Distribution of hotspot visitor counts. Spot counts for (A) simulated locations over 10 repetitions, and (B) observed locations. Example plot of observed field and the corresponding simulation (1 of 41). The red lines correspond to the hotspot threshold for this field (μ+2σ of the simulated location visitor counts). For this field the threshold is 4.047. Of the 498 locations in the simulated field 17 (3.41%) are hotspots (mean of 10 simulations). The observed field had 621 locations, of which 78 (12.5%) are hotspots, an increase of 258% over simulation.
(TIF)

S12 Fig Visualization of hotspots and hot tracks in 4 of 41 observed fields. Hotspots are indicated by black rectangles where the area is proportional to the number of unique visitors. Hot tracks are displayed in color with each color corresponding to a track. Tracks that do not visit a hot spot are shown in grey with the shades corresponding to individual tracks. Plots are a projection of a 3D space into the xy-plane. Overlapping hotspots indicate distinct z-coordinates.
(TIF)

S13 Fig A potential source of error is the dependence of the observed speed on the frame rate of observation. We test whether this confounding factor exists in our experiments by fitting a linear model to the mean speed for each of our seven binned microscope video frame rates vs the observed mean speed. Our frame delays range from 13 s to 20.7 s. The slope of the best MLE fit is 0.0013. The p-value is 0.66 and the *r*^2^ is 0.041. Together this suggests there is no relationship between frame rates and observed speed and that the observed speeds are not artifacts of the measuring rate.
(TIF)

S1 Movie Video of the simulation in progress. The video shows four instances of the efficiency simulation 1) An observed field, 2) Brownian motion simulation, 3) Power Law simulation, and 4) LogMCRW. Individual T cell tracks are variously colored according to track. Target DCs are green initially and turn cyan when encountered by a T cell.
(MP4)

S1 Table Extended step fit statistics. Table shows the Akaike information criterion evidence ratio (AIC E), applied to first 7 rows only; the corrected Akaike information criterion (AICc); negative log-likelihood (nlogl), Kolmogorov-Smirnov (KS), Anderson-Darling (AD), chi-squared (χ2), and Bayesian information criterion (BIC). Score ranking is in parentheses. Differences in BIC and AICc scores are less than 1:103 of the AICc score.
(DOCX)

S2 Table Extended speed fit statistics. Table shows the Akaike information criterion evidence ratio (AIC E), applied to first 7 rows only; the corrected Akaike information criterion (AICc); negative log-likelihood (nlogl), Kolmogorov-Smirnov (KS), Anderson-Darling (AD), chi-squared (χ2), and Bayesian information criterion (BIC). Score ranking is in parentheses. Differences in BIC and AICc scores are less than 1:103 of the AICc score.
(DOCX)

S3 Table Maximum likelihood estimated parameters and associated likelihood scores for steps calculated using a 30° threshold. The lognormal probability distribution is still the best fit when steps are calculated using a 30° rather than 15° threshold. Compare to Table 1 in the main text.
(DOCX)
