# Supplementary material for: Persistence and Adaptation in Immunity: T Cells Balance the Extent and Thoroughness of Search
Source: PLoS Comput Biol. 2016 Mar 18;12(3):e1004818. doi: 10.1371/journal.pcbi.1004818 (PMC4798282; doi:10.1371/journal.pcbi.1004818)
Supplement: S1 Text — (DOCX) [file pcbi.1004818.s001.docx]

Supplementary Methods

Fricke et al.

Persistence and adaptation in immunity: T cells balance the extent and thoroughness of search

**Extended Materials and Methods**

Analysis of T cell Tracks

Cell motility was analyzed with Imaris 6.0 (Bitplane AG, Zurich, Switzerland). Tracks with fewer than 3 time steps were removed from consideration. Tracks with total length or displacement from the start location less than 17 μm over the course of the observation were assumed to be non-motile and not included in analysis. The point sequences generated by Imaris were used to create position vectors joining adjacent cell locations (sample tracks Fig. S1). The Euclidean norm for each vector was calculated and divided by the time resolution to produce speeds. A nested ANOVA analysis [5] showed no differences between experiments replicated 41 times, using 17 mice, resulting in 159,746 positions over 5,077 T cell tracks. The combined track length for all cells was 34.8 cm and total observation time was 17 hours and 12 minutes. The mean track length is 34 positions with a median length of 21 and a max of 200. The maximum T cell velocity over all observations was 0.9 μm/s with a mean of 0.11 μm/s.

Microscopy fields with time resolution differences of less than 1 second were combined into groups so that mean squared displacement and vector autocorrelation could be calculated separately for groups with similar time resolution. Seven datasets were generated from the 41 observation fields. The autocorrelation plot, shown in Fig. 2E as a representative example, contains data from fields with time resolutions between 15 s and 16 s, and consists of 23,169 vectors from 537 tracks.

Speed measurements can be sensitive to the frame rate of observation. To determine whether the observed speed was influenced by the frame rate we fit a linear response model to frame delay and mean speed. The speed is not linearly correlated to the frame delay; as can be seen in Figure S13.

Distribution Fitting

Following Fisher [6] we use maximum likelihood estimation (MLE) to parameterize candidate PDFs. De Jager et al. provide details of the MLE method in their analysis of the motility patterns of mussels [7]. We fit probability model parameters using cumulative distribution functions (CDF), rather than by binning data which has been shown to bias conclusions about random walk distributions [8,9]. Software for fitting distributions was written in Matlab [10]. We examined a further 57 commonly used PDFs which were narrowed down to 17 for which we calculated negative log-likelihood scores (Tables S1 and S2) as candidate models.

A variety of probability distributions have relatively good statistical fit scores. Those that do are heavy-tailed, such as gamma and Weibull. Lognormal, while not the best fit is consistently high in the rankings for all goodness of fit measures (GoF), except the chi-squared test for step sizes. We choose to use the lognormal distribution for our simulations because it is relatively well understood mathematically and intuitively. See Fig. S4 for a comparison of the lognormal CDF to the gamma and Weibull CDFs. The lognormal distribution shows up repeatedly in biological contexts [11,12] so we have made it our representative for the heavy-tailed family of distributions.

Five PDF models for step length and speed were selected for analysis based on a combination of their negative log-likelihood scores, their importance in other biological processes, and their previous use in modeling T cell movement. Those models are lognormal, Maxwell, Gaussian, exponential, and power law PDFs. Our selection of the relative goodness of fit (GoF) of each candidate PDF to empirical data was evaluated using likelihood functions, Anderson-Darling (AD), Bayesian information criterion (BIC), corrected Akaiki Information Criterion (AICc), and the Kolmogorov-Smirnov (KS) test. We found all these measures to be in rank agreement and therefore present only the likelihood measure. We observe that for all GoF measures lognormal is ranked as a better fit to our observed step and speeds than the alternatives. In addition to track speeds, which we measure directly, we determined the distribution of step lengths, which is an essential element in the definition of Lévy walks. Since we image T cell motility frame by frame, we cannot track each cell continuously to ascertain step length. Instead, we define a *step* as a vector of T cell motion that does not deviate beyond 15^o^ from the original direction (see Fig. S8 for analysis of threshold dependency). We did not find any discrete angle thresholds and when we used 30^o^ as a cutoff angle to determine step length, we saw the same ranking in MLE fits (Table S3).

It is difficult to fit power laws in their simplest formulation,, with its infinite right variance, to necessarily finite biological systems. Additionally, power law behavior is often found in combination with other processes. Therefore, it is common to fit only a portion of the data to a power law, using formulations such as the generalized Pareto distribution, exponential cutoff, or generalized Lévy walk. In previous work [13] we modelled T cell search as a Lévy flight and found a good fit to T cell motion using the generalized Pareto distribution. Following Clauset, Shalizi, and Newman [9], we fit power laws using MLE and with the power law PDF: , where *x*_min_ is the smallest observed value, *P*(*x*) is the probability of *x* occurring, and is the estimated parameter. We used the *x*_min_ value with the best KS score of all possible choices as an estimator of the beginning of a power law tail. The percentage of positions in a track in the power law tail gives us a measure of the quality of the power law fit. Using this measure we show that a power law fit to the population of observed steps excludes 94% of the data (Fig. 1F and H). This measure is also used to filter tracks in S3.

Autocorrelation and Cross-Correlations

Velocity autocorrelations were calculated following Qian [14] and Tarantino et al. [15]. The autocorrelation function, , is the ensemble mean for the *n-1* possible delay times given the *n* vectors defining a T cell track.

$$\mathrm{C}_{\text{auto}}(\Delta t)=\left\langle v(t_{i})\cdot v(t_{j})\right\rangle,\forall i,j:\Delta t=t_{i}-t_{j}\text{ where }t_{i},t_{j}\text{ are times.}$$

The result is a measure of how much T cell direction depends on previous directions as a function of time delay. Letting be the unit velocity vector at time *t* belonging to the *k^th^* path, we defined the cross-correlation function,$\mathrm{C}_{\text{cross}}$, to be:

$$\mathrm{C}_{\text{cross}}(p)=\left\langle v(p_{k}(t))\cdot v(p_{m}(t))\right\rangle,\forall k,m\text{ where }p_{k},p_{m}\text{ are T cell paths.}$$

This measures the step angle dependence between T cell paths at the same moment in time, that is, a measure of drift due to global effects on the observation field.

Mean Squared Displacement

Mean squared displacement (MSD) coefficients, commonly called the *α* exponent were calculated using least-squares polynomial fit by numerically solving the associated Vandermonde matrix [16] and fit quality assessed with the *r*^2^ measure. Parametric and linear fits were also made to mean displacement. In Fig. 1A we present only the first 10 minutes of observation at which point the curve reaches its first stationary inflection which is indicative of unconstrained motion and therefore appropriate for determining *α*. In addition, in this study few tracks persist beyond 10 minutes and so the MSD signal also becomes dominated by noise (Fig 1A top and Fig. S9).

Heterogeneity

We used mixed Gaussian clustering [17] to investigate whether there is heterogeneity the distribution of track speeds among T cell tracks with different track mean speeds. The mean speed and variance in tracks is shown in both Fig. 2 and in Fig. S7. We further tested for heterogeneity by comparing track speed skew (Fig. 4) and AIC evidence ratios as a function of mean speed (data not shown).

Search Efficiency Simulation

We built a simulation to test how different movement patterns affect the efficiency with which T cells encounter DC targets, implemented as a continuous (floating-point) 3D model written in C++. Boost libraries (*59*) were used to generate variates drawn from model PDFs. Because the clustering and density of targets can influence which movement types are most efficient, we replicated the estimated density of DCs and varied the degree of clustering in our simulations.

Beltman et al. report a DC density in lymph node T cell zones of 2-5% [11,18]. We use this to calculate a target DC density of 3.17×10^-5^ targets/μm^3^. Our observed fields have an average volume of 6.3×10^6^ μm^3^. We scale the number of targets as a function of field volume in order to maintain the same target density between simulation fields. DCs were clustered into groups of 10 and were uniformly distributed within spheres defining a cluster. By varying the sphere radius, we controlled the degree of clustering from uniform to highly clustered. A 3D version of the Hopkins statistic [19] was used to measure the resulting non-uniformity of target placement (Tables 3 and 4). In the Hopkins statistic scores range from 0 to 0.5 where 0 is highly clustered and 0.5 indicates no clustering.

T cell tracks were observed and recorded as 3D coordinate sequences within a bounding box defined by the visible section of the *ex vivo* lymph node. Idealized models (Brownian, CRW, Power Law, etc.) of search were parameterized by the speeds and turning angles estimated from observation (see Distribution Fitting). Searchers in the idealized model start at the same initial positions as the observed T cells, and exist in a volume equal to the observed field volume. Candidate search patterns were generated for each of the 41 observation fields. For sample visualization of each idealized model search as well as the observed see Fig. S10.

Similar to [20] our measure of efficiency, $\mathrm{E}(k,\text{Obs})$, for a combination of *n* observed tracks (Obs), search strategy, and target distribution is the number of targets discovered,$\mathrm{F}(k)$,by each searcher, *k*, divided by the time expended, $\mathrm{T}(k)$, by the *k^th^* track. The distance covered by track *k* is$\mathrm{D}(k)$, and the total distance covered by observed tracks is$\mathrm{D}(\text{Obs})$. L is the property that the total distance expended by searchers does not exceed the distance expended by observed T cells.

$$\mathrm{E}(k,\text{Obs})=\frac{\sum_{k=1}^{\mathrm{L}(k)} \mathrm{F}(k)}{\sum_{k=1}^{\mathrm{L}(k)} \mathrm{T}(k)},\text{ where }\mathrm{L}(k)=\sum_{i=1}^{k} \mathrm{D}(i)<\mathrm{D}(\text{Obs}).$$

Efficiency measure E is the number of targets found divided by the sum of the time used by searchers. Since we modelled walks rather than flights (i.e. speeds are finite) the sum of $\mathrm{D}(k)$for all simulated tracks$k$was limited to the total distance travelled by observed T cells. Therefore the average velocity of the population of searchers is kept within the observed range. In the limit where the field is saturated with targets, the efficiency of unique contacts would be the swept volume of each track. Based on an assumed radii of 5 μm for DCs and T cell, targets were marked as discovered if a searcher track passed within 10 μm of a target point. We define two versions of F(*k*), one that increments its output value only when a target was not previously detected by searcher, *k*, and another that increments for all targets found. These two versions of F(*k*) allow us to record unique contacts and total contacts (Fig. 4).

The simulation measures the target encounter rate and determines, using the Mann-Whiney test, whether the candidate search models’ search efficiency is significantly different from that observed in T cells. We use the Mann-Whitney test because the observed and simulated distribution of efficiencies is non-Gaussian. Bonferoni correction [21] was used to adjust for the minimum p-value that can be called significant. Simulations were replicated 100 times per field, producing 4,200 efficiency data points for each search model. The entire process was repeated 10 times in order to generate confidence intervals for the simulation; in all this results in 41,000 efficiency samples.

Identifying Hotspots and Hot Tracks

In order to test whether the environment within LNs influences T cell movement we extend an analysis begun in [22]. To determine hotspots, we use the LogMCRW simulation as a null model. We discretize the LN into cubes with 20 μm edges (about twice the diameter of a T cell). We record the number of times a location is visited by unique T cells in simulation (repeated 10 times). We use a 2σ (standard deviation) threshold for determining which locations are visited particularly frequently than expected and call these hotspots (threshold indicated by red line in Fig. S11). This is repeated for each of the 41 individual observational fields. We then determine hotspots for observed data as locations visited more frequently than the threshold set by the null model that corresponds to the individual observation field (graphically shown in Fig. S12). All other locations are called cold spots.

We define hot tracks to be T cell tracks that visit hotspots and cold tracks to be T cell tracks that do not. We also examine the number of visits by hot tracks to cold spots and hotspots.

**References and Notes**

1. Matheu MP, Parker I, Cahalan MD. Dissection and 2-photon imaging of peripheral lymph nodes in mice. J Vis Exp JoVE. MyJoVE Corporation; 2007;

2. Allenspach EJ, Cullinan P, Tong J, Tang Q, Tesciuba AG, Cannon JL, et al. ERM-dependent movement of CD43 defines a novel protein complex distal to the immunological synapse. Immunity. Elsevier; 2001;15: 739–50. Available: http://www.ncbi.nlm.nih.gov/pubmed/11728336

3. Huang JH, Cárdenas-Navia LI, Caldwell CC, Plumb TJ, Radu CG, Rocha PN, et al. Requirements for T lymphocyte migration in explanted lymph nodes. J Immunol. Am Assoc Immnol; 2007;178: 7747–7755.

4. Viswanathan GM, Raposo EP, Bartumeus F, Catalan J, da Luz MGE. Necessary criterion for distinguishing true superdiffusion from correlated random walk processes. Phys Rev E. American Physical Society; 2005;72: 11111. doi:10.1103/PhysRevE.72.011111

5. Letendre K, Donnadieu E, Moses ME, Cannon JL. Bringing Statistics Up To Speed With Data in Analysis of Lymphocyte Motility. PLoS One. 2015;

6. Fisher RA. Theory of statistical estimation. Mathematical Proceedings of the Cambridge Philosophical Society. Cambridge Univ Press; 1925.

7. De Jager M, Bartumeus F, Kölzsch A, Weissing FJ, Hengeveld GM, Nolet BA, et al. How superdiffusion gets arrested: ecological encounters explain shift from Lévy to Brownian movement. Proc R Soc B Biol Sci. The Royal Society; 2014;281: 20132605.

8. Goldstein ML, Morris SA, Yen GG. Problems with fitting to the power-law distribution. Eur Phys J B-Condensed Matter Complex Syst. Springer; 2004;41: 255–258.

9. Clauset A, Shalizi CR, Newman MEJ. Power-law distributions in empirical data. SIAM Rev. SIAM; 2009;51: 661–703.

10. MATLAB with Statistics Toolbox Release 2014a. Natick, Massachusetts, United States: The MathWorks, Inc;

11. Beltman JB, Marée AFM, Lynch JN, Miller MJ, de Boer RJ. Lymph node topology dictates T cell migration behavior. J Exp Med. 2007;204: 771–80. doi:10.1084/jem.20061278

12. Furusawa C, Suzuki T, Kashiwagi A, Yomo T, Kaneko K. Ubiquity of log-normal distributions in intra-cellular reaction dynamic. arXiv Prepr q-bio/0503040. 2005;

13. Fricke GM, Asperti-Boursin F, Hecker J, Cannon J, Moses M. From Microbiology to Microcontrollers: Robot Search Patterns Inspired by T Cell Movement. Advances in Artificial Life, ECAL. 2013. pp. 1009–1016.

14. Qian H, Sheetz MP, Elson EL. Single particle tracking. Analysis of diffusion and flow in two-dimensional systems. Biophys J. Elsevier; 1991;60: 910–921.

15. Tarantino N, Tinevez J-Y, Crowell EF, Boisson B, Henriques R, Mhlanga M, et al. TNF and IL-1 exhibit distinct ubiquitin requirements for inducing NEMO–IKK supramolecular structures. J Cell Biol. Rockefeller Univ Press; 2014;204: 231–245.

16. Von Mises R, Geiringer H. Mathematical theory of probability and statistics. Academic Press New York; 1964.

17. McLachlan G, Peel D. Finite mixture models. John Wiley & Sons; 2004.

18. Beltman JB, Marée AFM, de Boer RJ. Analysing immune cell migration. Nat Rev Immunol. Nature Publishing Group; 2009;9: 789–98. doi:10.1038/nri2638

19. Hopkins B, Skellam JG. A new method for determining the type of distribution of plant individuals. Ann Bot. Annals Botany Co; 1954;18: 213–227.

20. James A, Pitchford JW, Plank MJ. Efficient or inaccurate? Analytical and numerical modelling of random search strategies. Bull Math Biol. 2010;72: 896–913. doi:10.1007/s11538-009-9473-z

21. Bland JM, Altman DG. Multiple significance tests: the Bonferroni method. Bmj. BMJ; 1995;310: 170.

22. Fricke GM, Hecker JP, Black SR, Cannon JL, Moses ME, Hecker JP, et al. Distinguishing Adaptive Search From Random Search in Robots and T cells Categories and Subject Descriptors. GECCO ’15 Proceedings of the 2015 on Genetic and Evolutionary Computation Conference. ACM; 2015. pp. 105–112. doi:10.1145/2739480.2754794
