# Supplementary figures and images for: Persistence and Adaptation in Immunity: T Cells Balance the Extent and Thoroughness of Search
Source: PLoS Comput Biol. 2016 Mar 18;12(3):e1004818. doi: 10.1371/journal.pcbi.1004818 (PMC4798282; doi:10.1371/journal.pcbi.1004818)

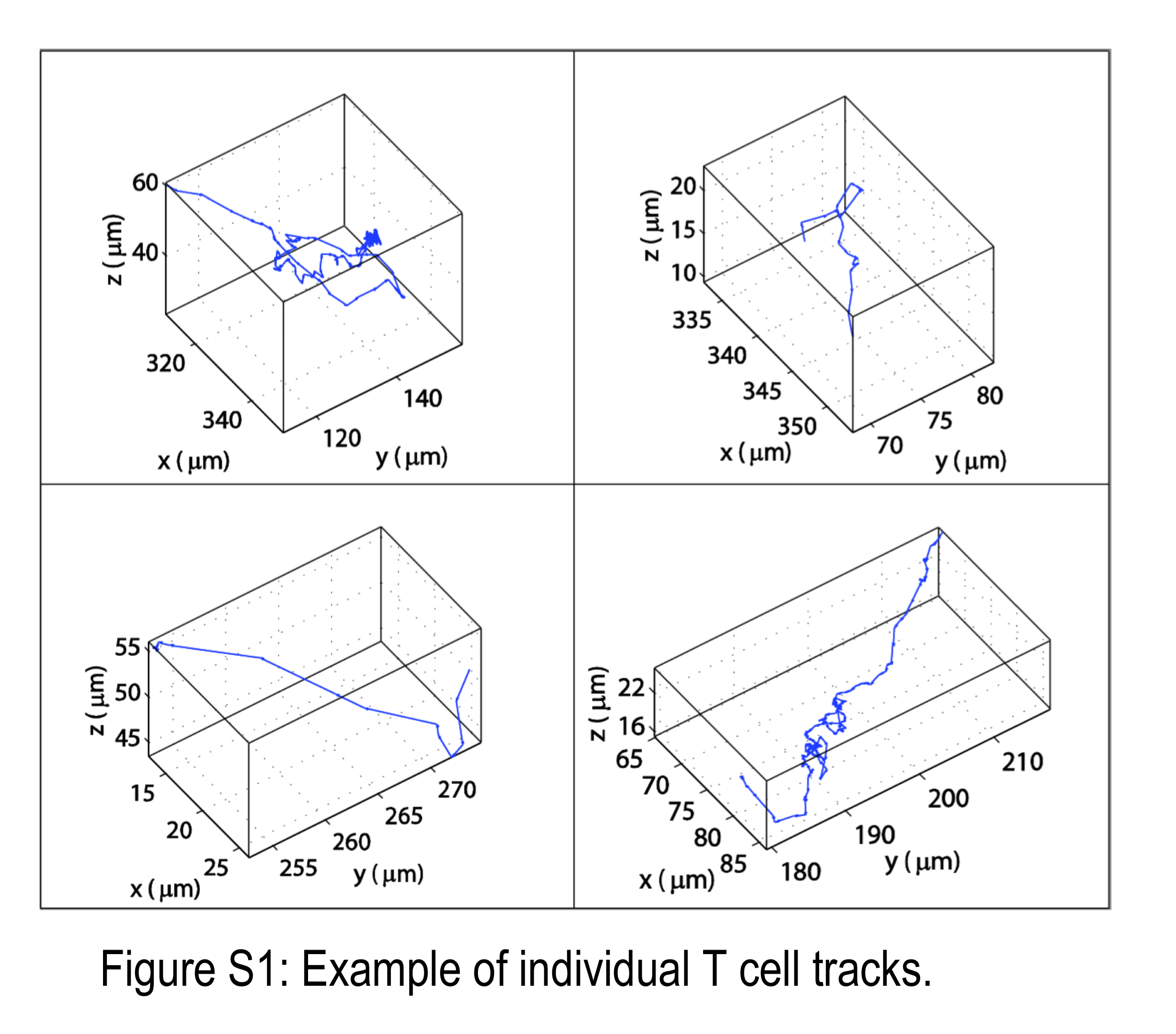

Supplement: S1 Fig — (TIF) [file pcbi.1004818.s002.tif]

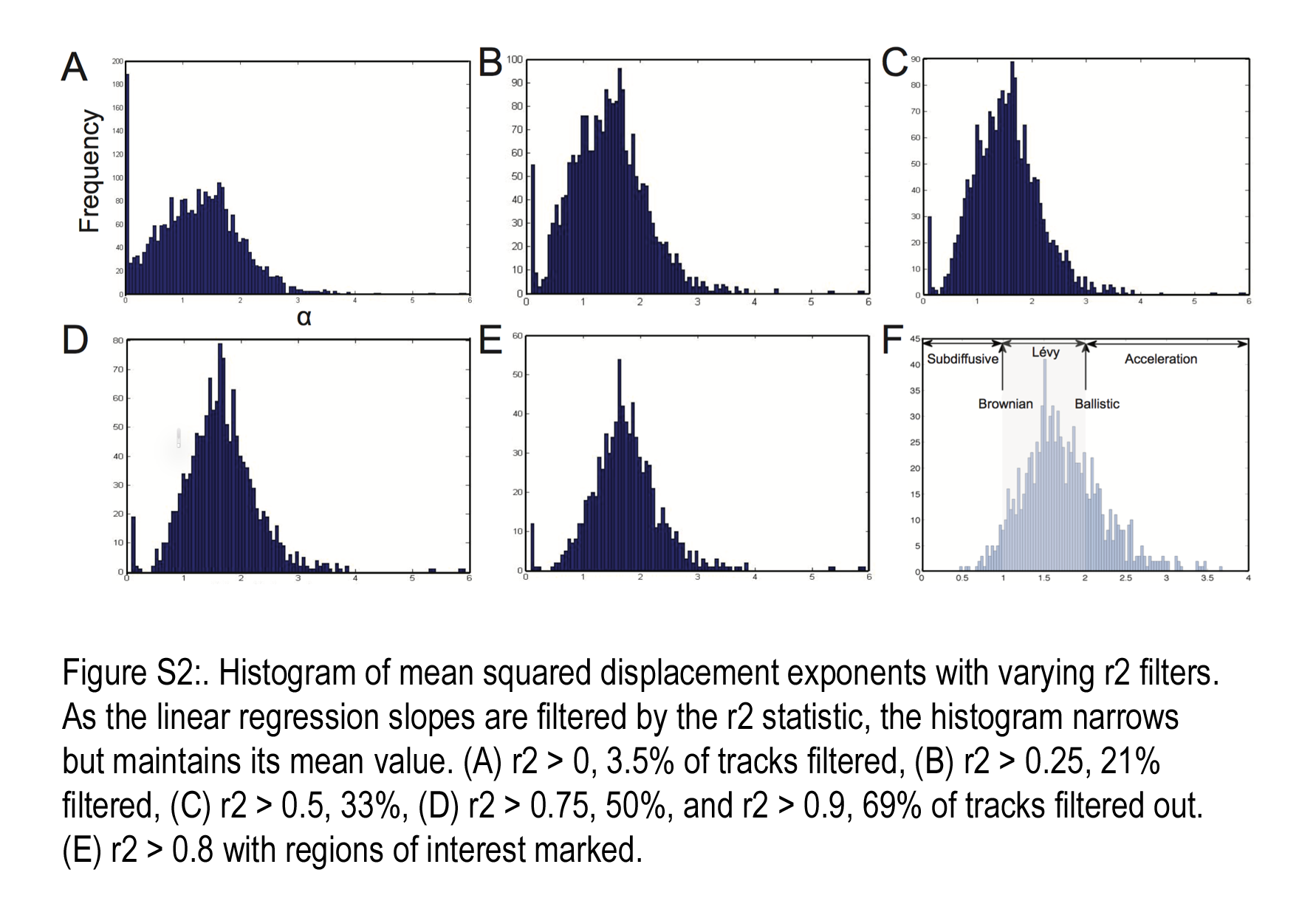

Supplement: S2 Fig — As the linear regression slopes are filtered by the r2 statistic, the histogram narrows but maintains its mean value. (A) r2 > 0, 3.5% of tracks filtered, (B) r2 > 0.25, 21% filtered, (C) r2 > 0.5, 33%, (D) r2 > 0.75, 50%, and r2 > 0.9, 69% of tracks filtered out. (E) r2 > 0.8 with regions of interest marked. (TIF) [file pcbi.1004818.s003.tif]

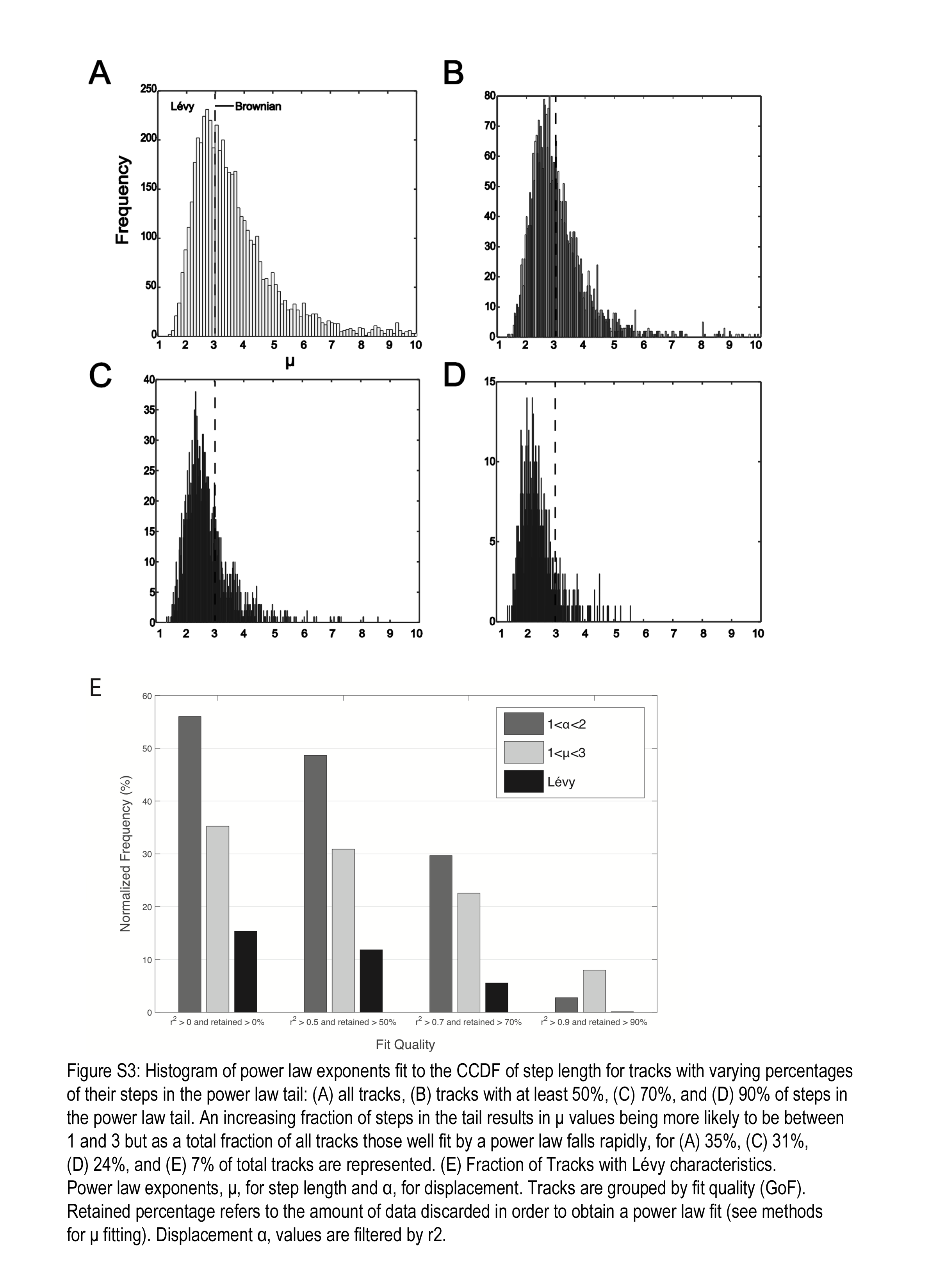

Supplement: S3 Fig — Histogram of power law exponents fit to the CCDF of step length for tracks with varying percentages of their steps in the power law tail: (A) all tracks, (B) tracks with at least 50%, (C) 70%, and (D) 90% of steps in the power law tail. An increasing fraction of steps in the tail results in μ values being more likely to be between 1 and 3 but as a total fraction of all tracks those well fit by a power law falls rapidly, for (A) 35%, (C) 31%, (D) 24%, and (E) 7% of total tracks are represented. (E) Fraction of Tracks with Lévy characteristics. Power law exponents, μ, for step length and α, for displacement. Tracks are grouped by fit quality (GoF). Retained percentage refers to the amount of data discarded in order to obtain a power law fit (see methods for μ fitting). Displacement α, values are filtered by r2. (TIF) [file pcbi.1004818.s004.tif]

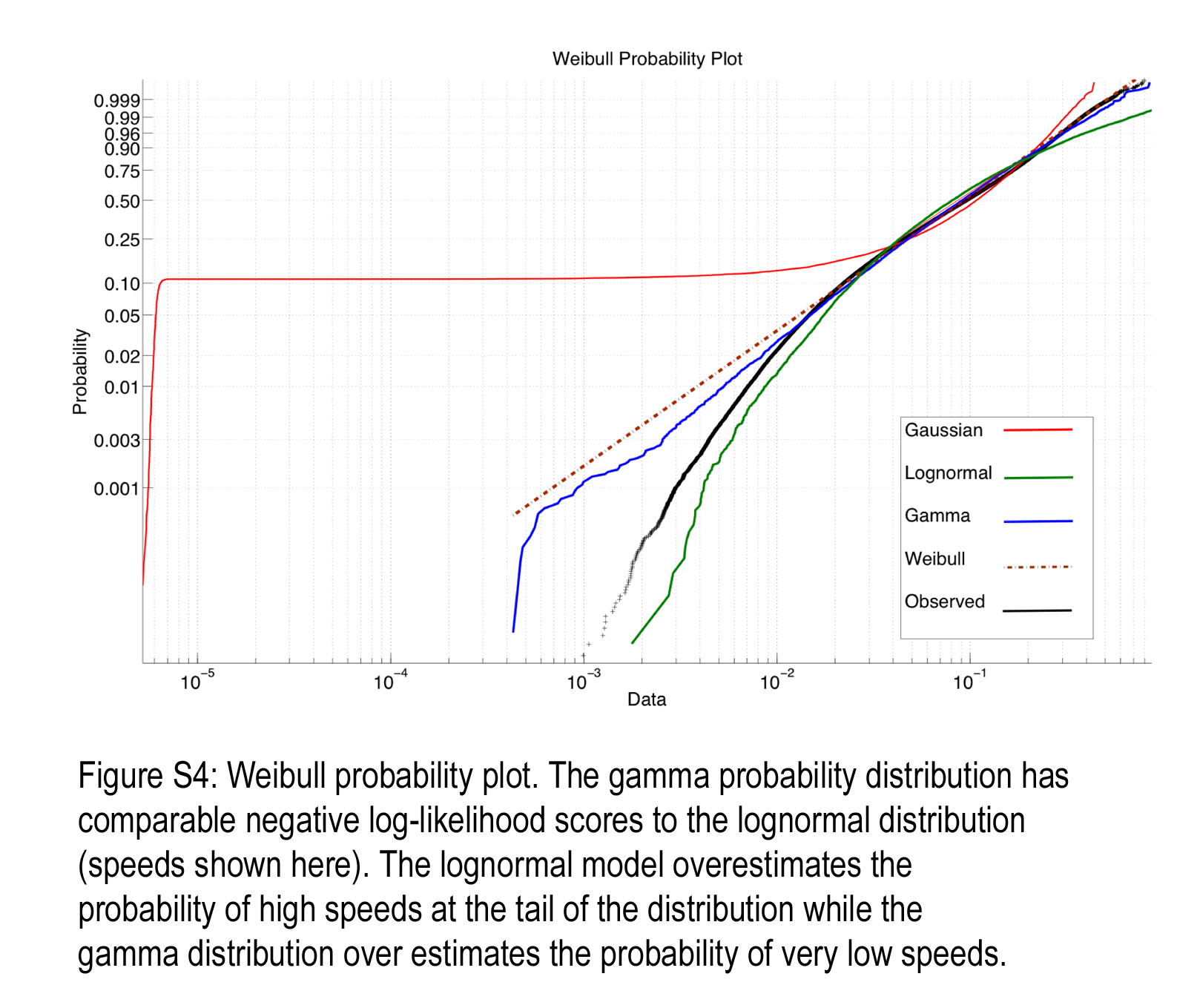

Supplement: S4 Fig — The gamma probability distribution has comparable negative log-likelihood scores to the lognormal distribution (speeds shown here). The lognormal model overestimates the probability of high speeds at the tail of the distribution while the gamma distribution over estimates the probability of very low speeds. (TIF) [file pcbi.1004818.s005.tif]

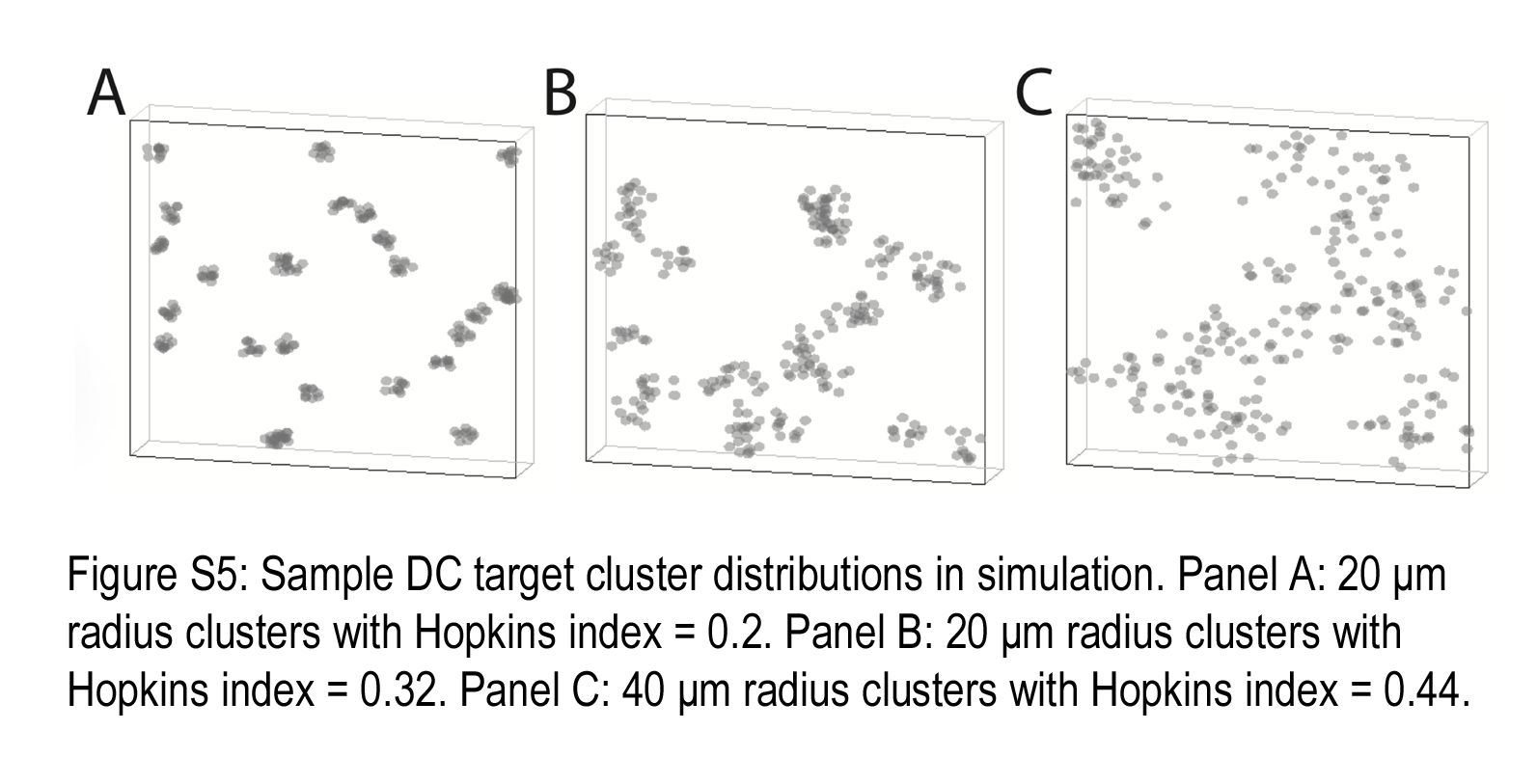

Supplement: S5 Fig — Panel A: 20 μm radius clusters with Hopkins index = 0.2. Panel B: 20 μm radius clusters with Hopkins index = 0.32. Panel C: 40 μm radius clusters with Hopkins index = 0.44. (TIF) [file pcbi.1004818.s006.tif]

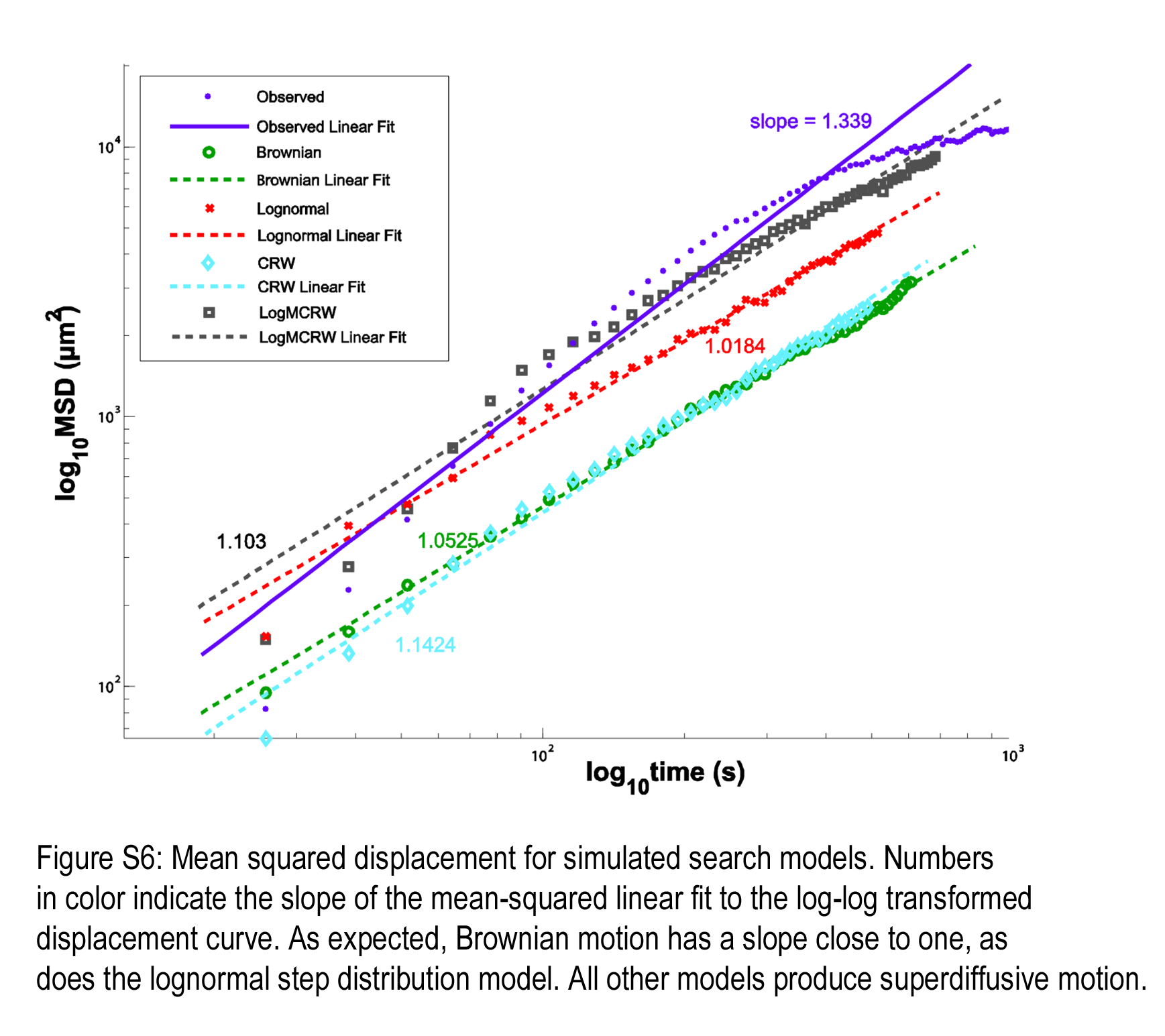

Supplement: S6 Fig — Numbers in color indicate the slope of the mean-squared linear fit to the log-log transformed displacement curve. As expected, Brownian motion has a slope close to one, as does the lognormal step distribution model. All other models produce superdiffusive motion. (TIF) [file pcbi.1004818.s007.tif]

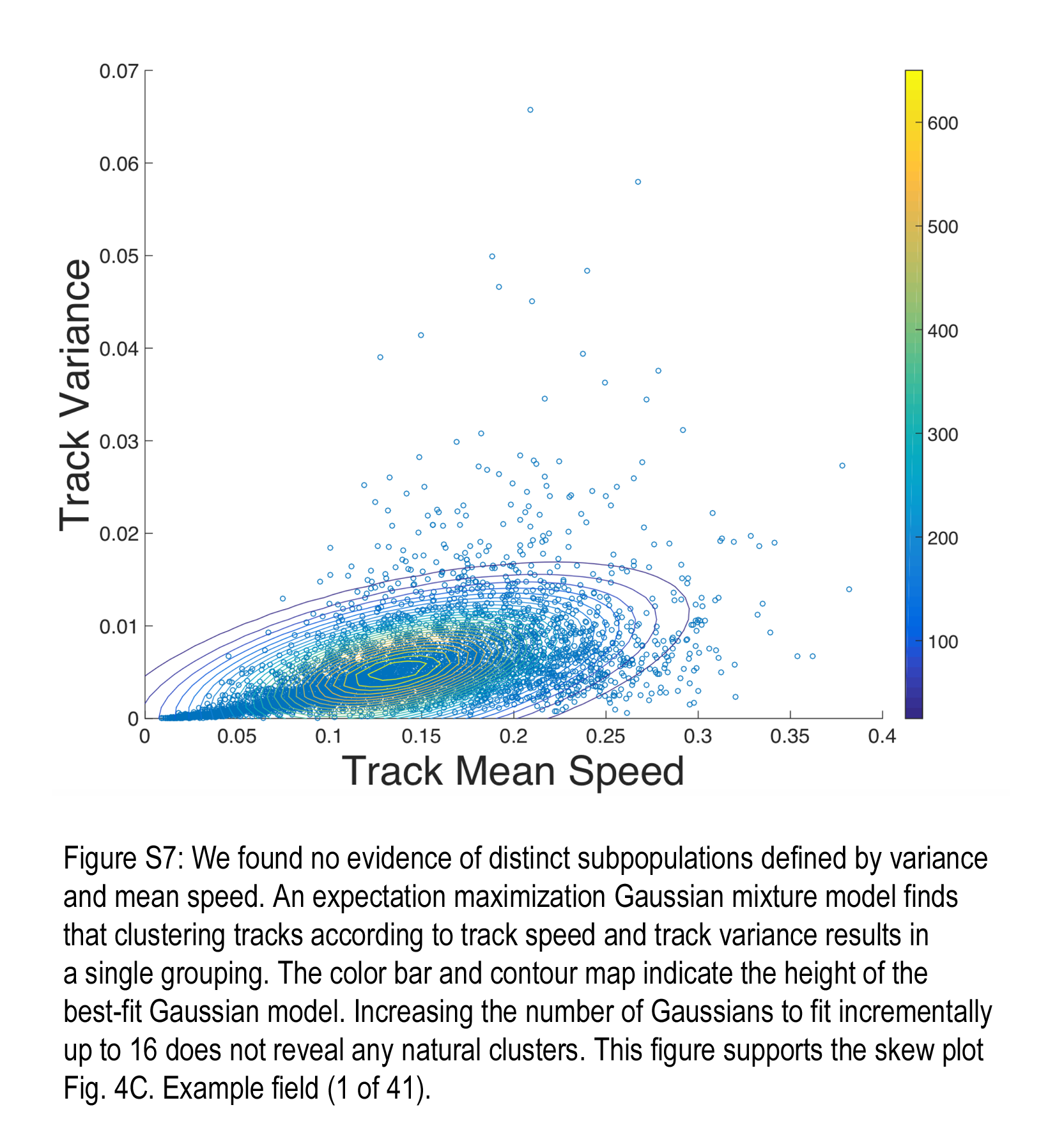

Supplement: S7 Fig — An expectation maximization Gaussian mixture model finds that clustering tracks according to track speed and track variance results in a single grouping. The color bar and contour map indicate the height of the best-fit Gaussian model. Increasing the number of Gaussians to fit incrementally up to 16 does not reveal any natural clusters. This figure supports the skew plot Fig 4C. Example field (1 of 41). (TIF) [file pcbi.1004818.s008.tif]

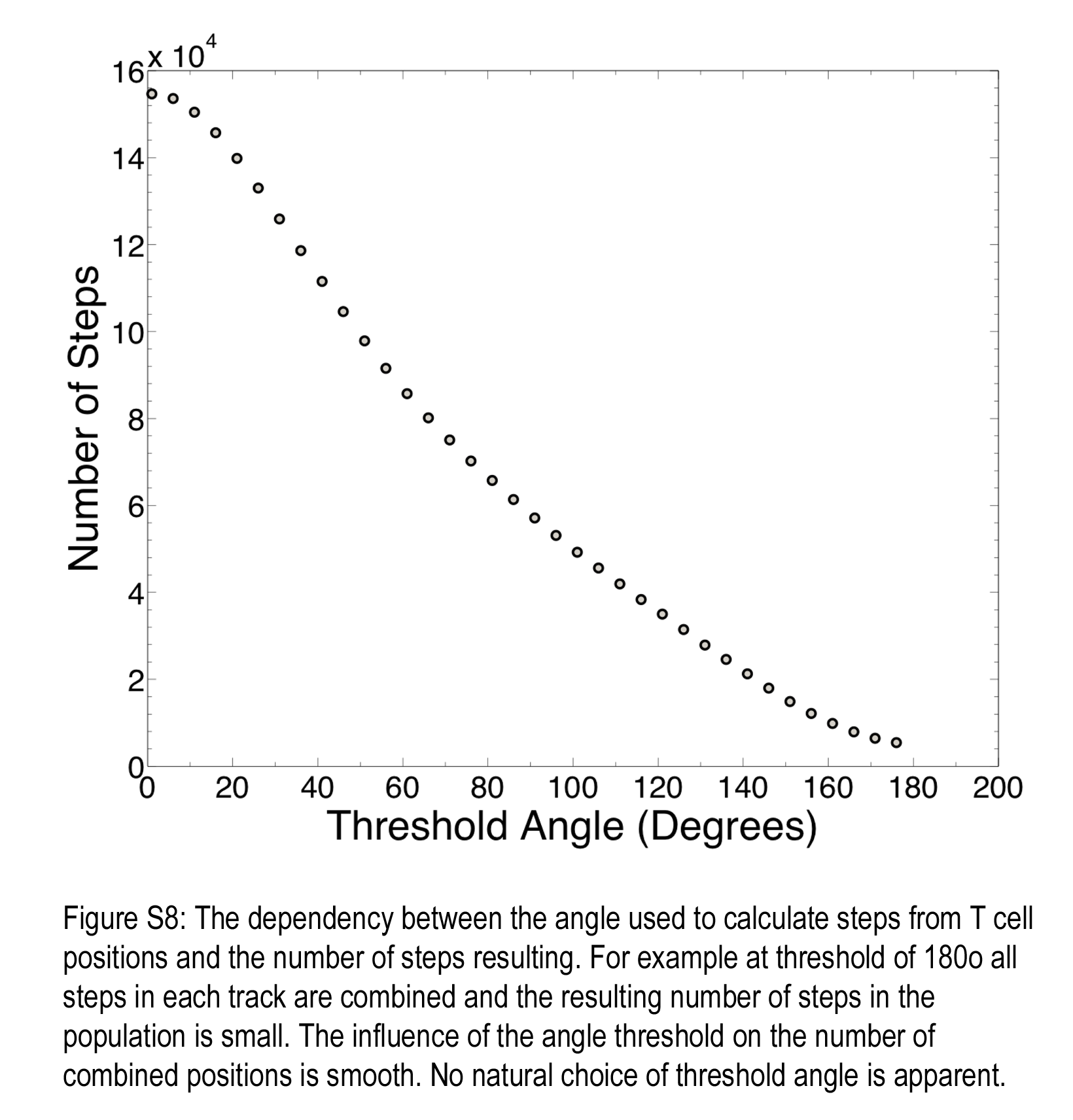

Supplement: S8 Fig — For example at threshold of 180° all steps in each track are combined and the resulting number of steps in the population is small. The influence of the angle threshold on the number of combined positions is smooth. No natural choice of threshold angle is apparent. (TIF) [file pcbi.1004818.s009.tif]

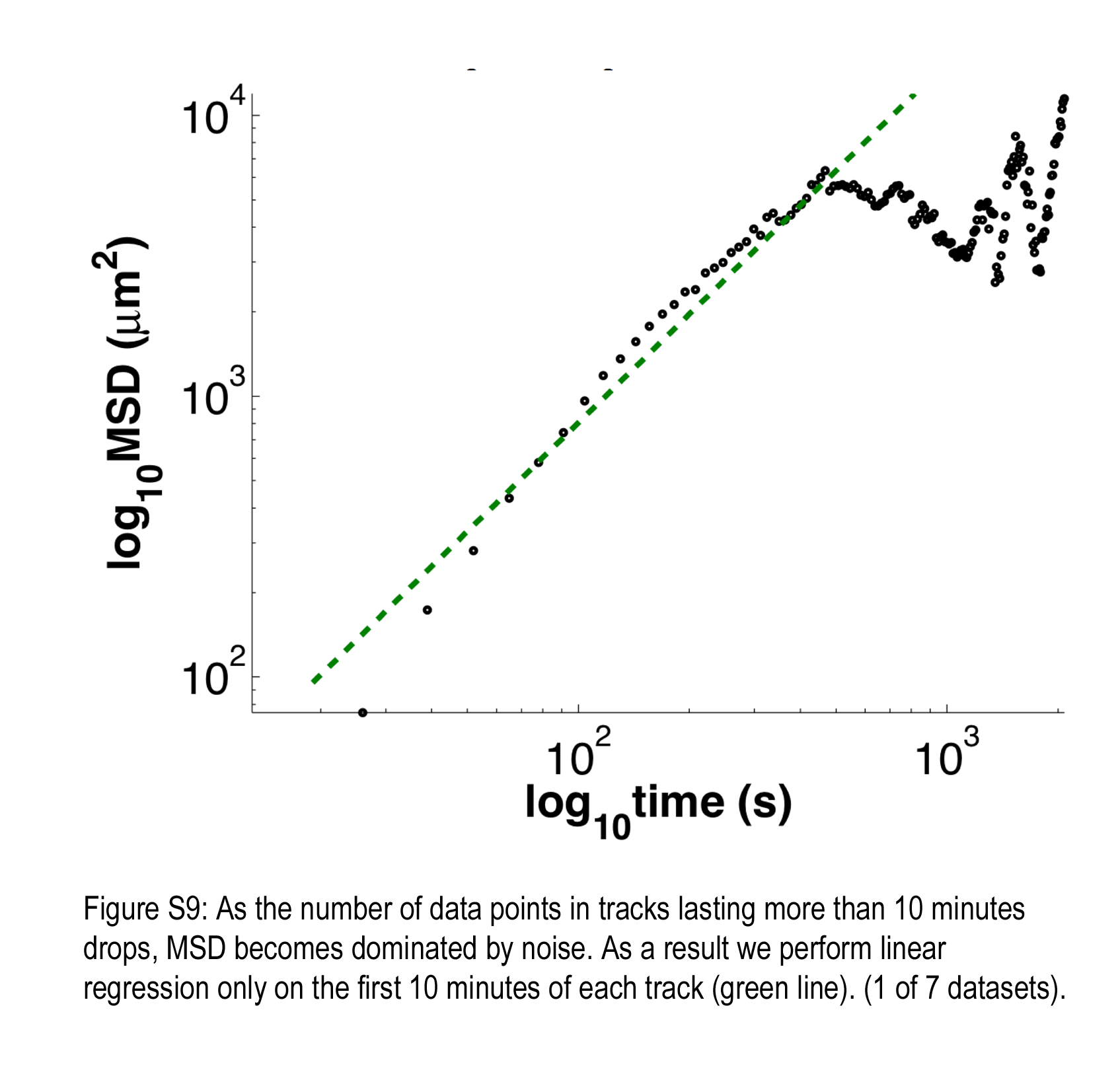

Supplement: S9 Fig — As a result we perform linear regression only on the first 10 minutes of each track (green line). (1 of 7 datasets). (TIF) [file pcbi.1004818.s010.tif]

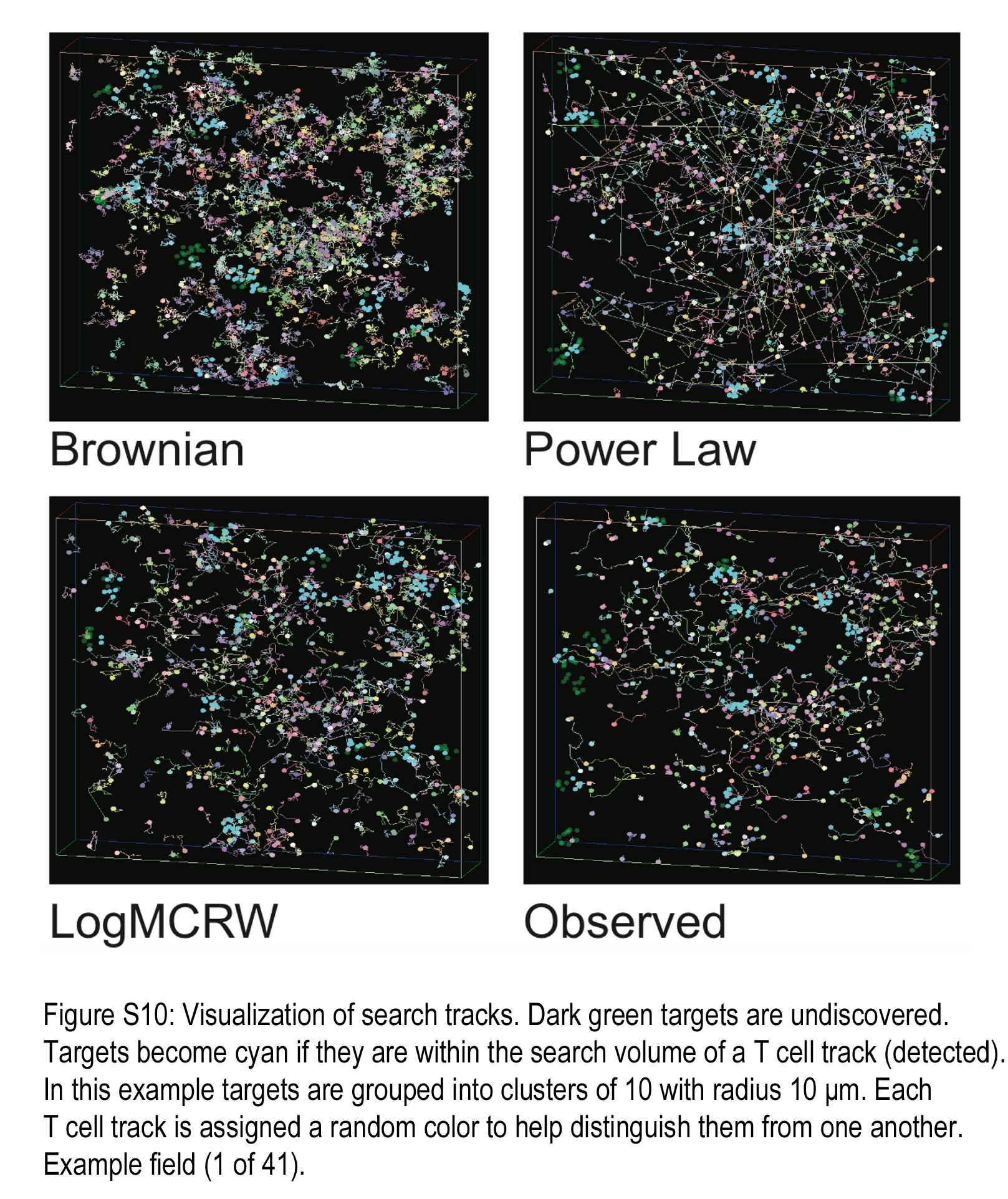

Supplement: S10 Fig — Dark green targets are undiscovered. Targets become cyan if they are within the search volume of a T cell track (detected). In this example targets are grouped into clusters of 10 with radius 10 μm. Each T cell track is assigned a random color to help distinguish them from one another. Example field (1 of 41). (TIF) [file pcbi.1004818.s011.tif]

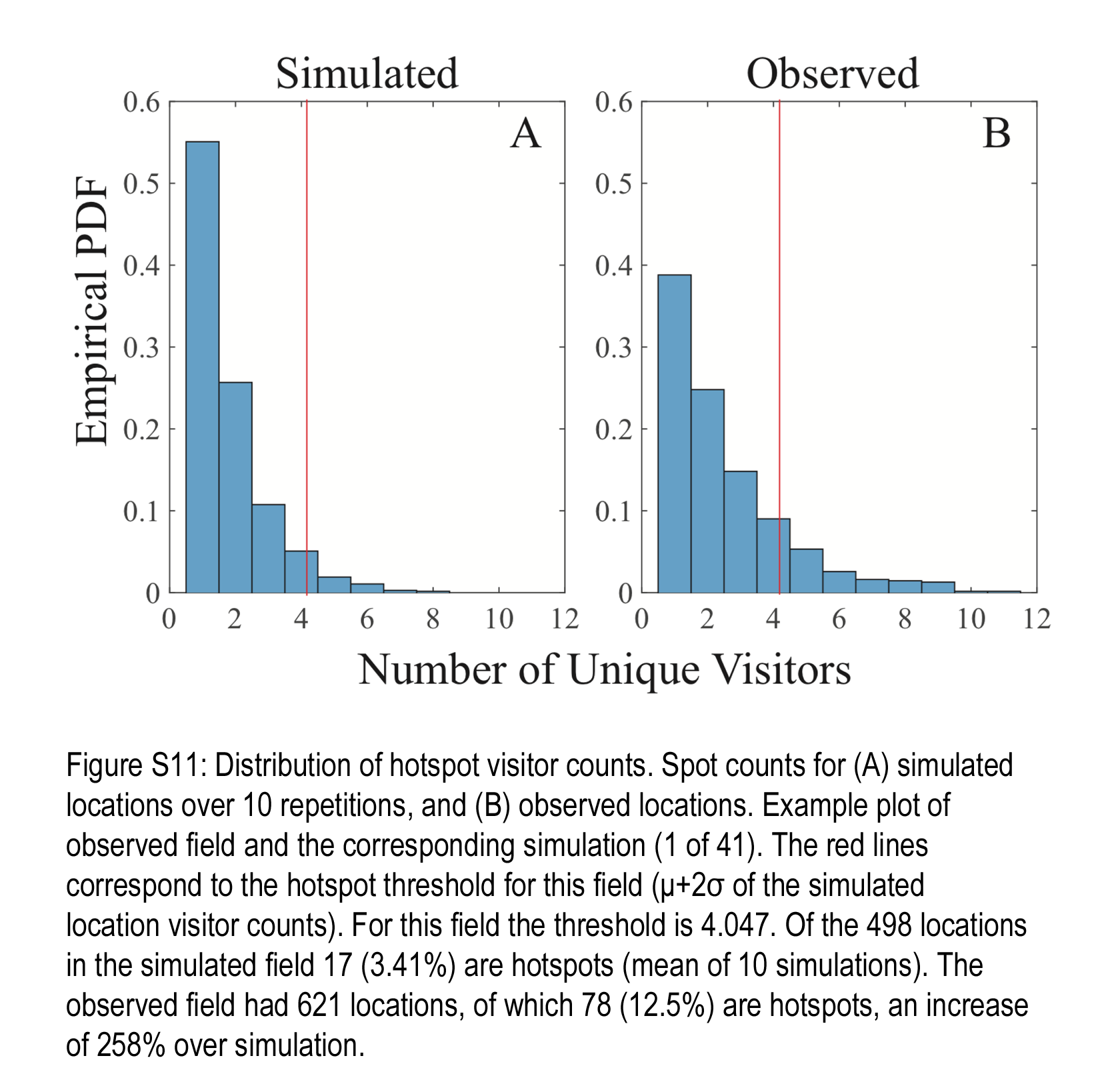

Supplement: S11 Fig — Spot counts for (A) simulated locations over 10 repetitions, and (B) observed locations. Example plot of observed field and the corresponding simulation (1 of 41). The red lines correspond to the hotspot threshold for this field (μ+2σ of the simulated location visitor counts). For this field the threshold is 4.047. Of the 498 locations in the simulated field 17 (3.41%) are hotspots (mean of 10 simulations). The observed field had 621 locations, of which 78 (12.5%) are hotspots, an increase of 258% over simulation. (TIF) [file pcbi.1004818.s012.tif]

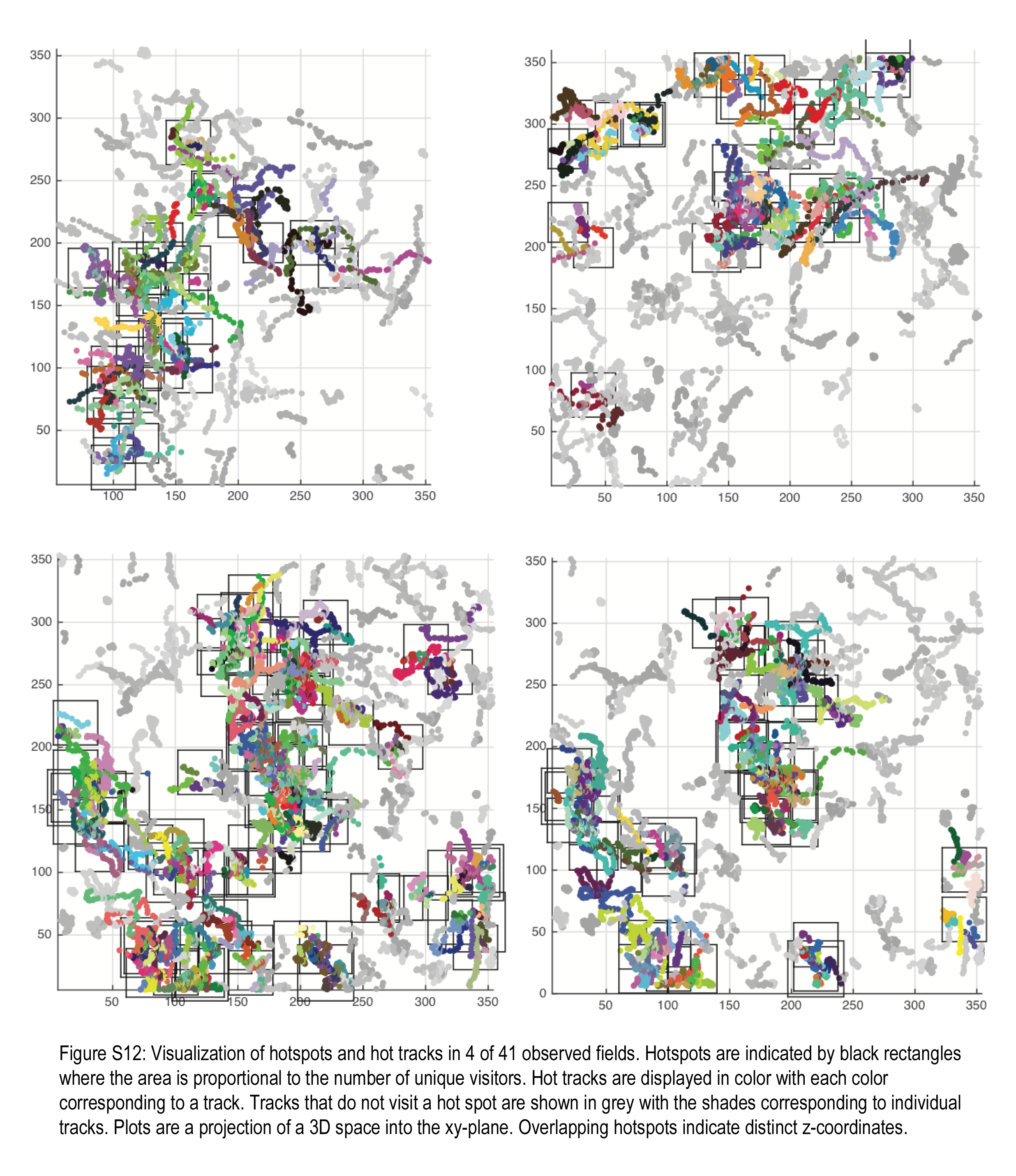

Supplement: S12 Fig — Hotspots are indicated by black rectangles where the area is proportional to the number of unique visitors. Hot tracks are displayed in color with each color corresponding to a track. Tracks that do not visit a hot spot are shown in grey with the shades corresponding to individual tracks. Plots are a projection of a 3D space into the xy-plane. Overlapping hotspots indicate distinct z-coordinates. (TIF) [file pcbi.1004818.s013.tif]

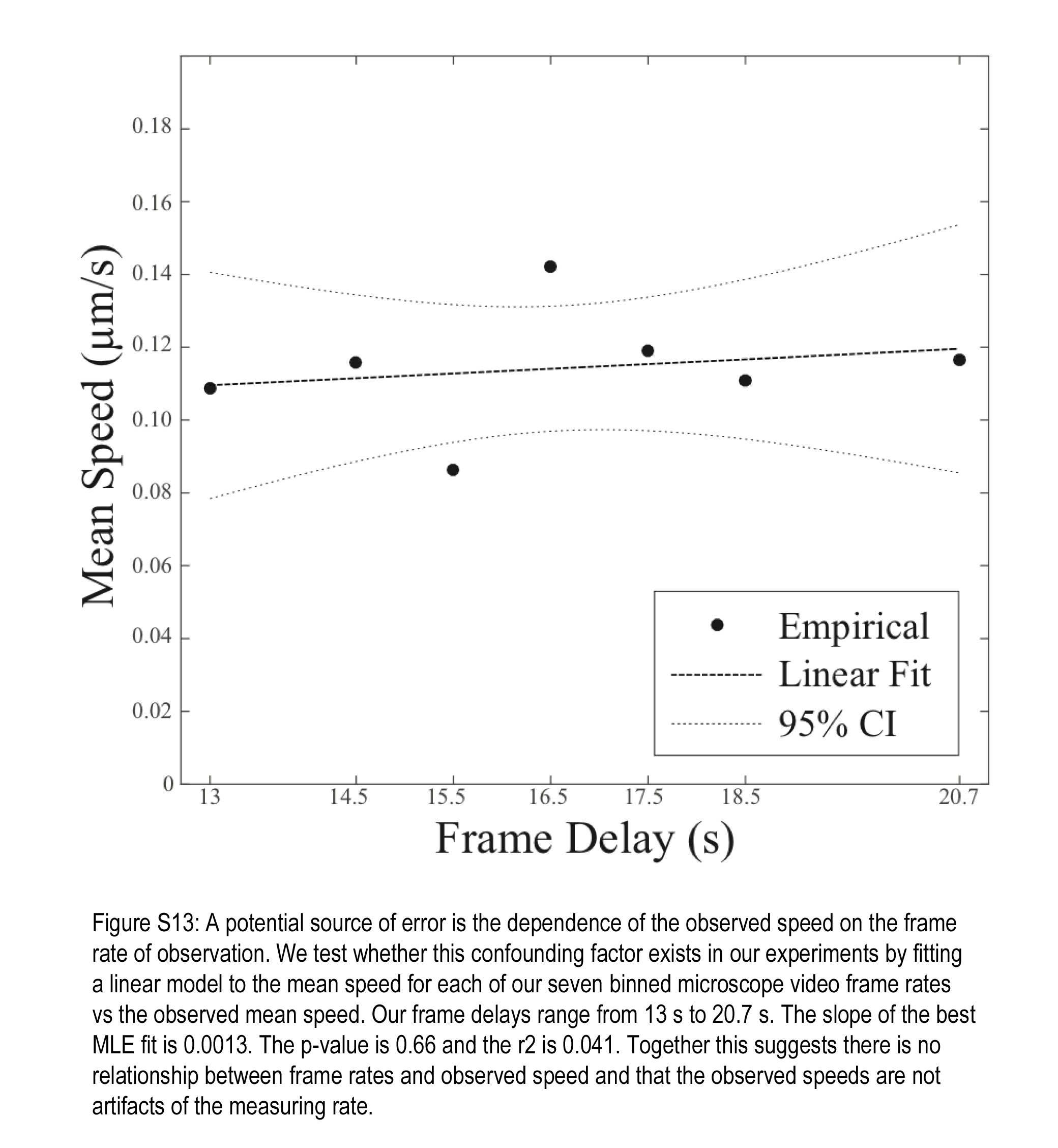

Supplement: S13 Fig — We test whether this confounding factor exists in our experiments by fitting a linear model to the mean speed for each of our seven binned microscope video frame rates vs the observed mean speed. Our frame delays range from 13 s to 20.7 s. The slope of the best MLE fit is 0.0013. The p-value is 0.66 and the r2 is 0.041. Together this suggests there is no relationship between frame rates and observed speed and that the observed speeds are not artifacts of the measuring rate. (TIF) [file pcbi.1004818.s014.tif]
