# Supplementary material for: Persistence and Adaptation in Immunity: T Cells Balance the Extent and Thoroughness of Search
Source: PLoS Comput Biol. 2016 Mar 18;12(3):e1004818. doi: 10.1371/journal.pcbi.1004818 (PMC4798282; doi:10.1371/journal.pcbi.1004818)
Supplement: S1 Table — Table shows the Akaike information criterion evidence ratio (AIC E), applied to first 7 rows only; the corrected Akaike information criterion (AICc); negative log-likelihood (nlogl), Kolmogorov-Smirnov (KS), Anderson-Darling (AD), chi-squared (χ2), and Bayesian information criterion (BIC). Score ranking is in parentheses. Differences in BIC and AICc scores are less than 1:103 of the AICc score. (DOCX) [file pcbi.1004818.s016.docx]

**Supplemental Table 1**

**Extended Goodness of Fit (GoF) Statistics**

| **Distribution** | **AIC E** | **AICc (×10^5^)** | **nlogl (×10^5^)** | **KS** | **AD (×10^3^)** | **χ^2^ (×10^3^)** | **BIC (×10^5^)** |
| --- | --- | --- | --- | --- | --- | --- | --- |
| **Lognormal** | 1 | 5.29 (2) | 2.65 (2) | 0.06 (4) | 1.50 (2) | 8.06 (7) | 5.29 (2) |
| **Gamma** | 0 | 5.34 (3) | 2.67 (3) | 0.04 (2) | 0.57 (1) | 4.15 (4) | 5.34 (3) |
| **Gaussian** | 0 | 6.72 (10) | 3.36 (10) | 0.10 (5) | 3.57 (4) | 26.56 (9) | 6.72 (10) |
| **Power Law** | 0 | 9.16 (15) | 4.58 (15) | 0.33 (8) | 38.02 (6) | 154.68 (11) | 9.16 (15) |
| **Maxwell** | 0 | 8.04 (14) | 4.02 (13) |  |  |  | 8.04 (14) |
| **Exponential** | 0 | 7.34 (11) | 3.67 (11) | 0.09 (7) | 2.38 (3) | 13.13 (8) | 7.34 (11) |
| **Gen. Pareto** | 0 | 5.48 (5) | 2.74 (5) | 0.04 (2) | 19.89 (5) |  | 5.48 (5) |
| **Fatigue** |  | 4.98 (1) | 2.49 (1) | 0.05 (3) | 597.0 (9) | 2.95 (2) | 4.98 (1) |
| **Nakagami** |  | 5.41 (4) | 2.7 (4) |  |  |  | 5.41 (4) |
| **Weibull** |  | 5.51 (6) | 2.76 (6) | 0.05 (3) | 1117.1 (11) | 6.57 (6) | 5.51 (6) |
| **Loglogistic** |  | 5.56 (7) | 2.78 (7) | 0.04 (2) | 442.86 (8) | 4.01 (3) | 5.56 (7) |
| **T-location**  **Scale** |  | 5.71 (8) | 2.86 (8) |  |  |  | 5.71 (8) |
| **Extreme Value** |  | 7.80 (13) | 3.9 (13) | 0.03 (1) | 278.92 (7) | 2.69 (1) | 7.80 (13) |
| **Inv. Gaussian** |  | 6.01 (9) | 3.04 (10) | 0.03 (1) | 696.67 (10) | 4.96 (5) | 6.01 (9) |
| **Logistic** |  | 7.73 (12) | 3.87 (12) |  |  |  | 7.73 (12) |
| **Rayleigh** |  | 10.06 (16) | 5.29 (16) | 0.17 (6) | 14565.0 (12) | 56.65 (10) | 10.06 (16) |
| **Rician** |  | 21.40 (17) | 10.71 (17) |  |  |  | 21.40 (17) |

**Table S1. Extended Step Fit Statistics.** Table shows the Akaike information criterion evidence ratio (AIC E), applied to first 7 rows only; the corrected Akaike information criterion (AICc); negative log-likelihood (nlogl), Kolmogorov-Smirnov (KS), Anderson-Darling (AD), chi-squared (χ^2^), and Bayesian information criterion (BIC). Score ranking is in parentheses. Differences in BIC and AICc scores are less than 1:10^3^ of the AICc score.
