# Supplementary material for: Persistence and Adaptation in Immunity: T Cells Balance the Extent and Thoroughness of Search
Source: PLoS Comput Biol. 2016 Mar 18;12(3):e1004818. doi: 10.1371/journal.pcbi.1004818 (PMC4798282; doi:10.1371/journal.pcbi.1004818)
Supplement: S2 Table — Table shows the Akaike information criterion evidence ratio (AIC E), applied to first 7 rows only; the corrected Akaike information criterion (AICc); negative log-likelihood (nlogl), Kolmogorov-Smirnov (KS), Anderson-Darling (AD), chi-squared (χ2), and Bayesian information criterion (BIC). Score ranking is in parentheses. Differences in BIC and AICc scores are less than 1:103 of the AICc score. (DOCX) [file pcbi.1004818.s017.docx]

**Supplemental Table 2**

| **Distribution** | **AIC E** | **AICc (×10^5^)** | **nlogl (×10^5^)** | **KS** | **AD (×10^3^)** | **χ ^2^ (×10^3^)** | **BIC** |
| --- | --- | --- | --- | --- | --- | --- | --- |
| **Lognormal** | 0 | -3.68 (5) | -1.84 (4) | 0.04 (5) | 784.54 (3) | 8.06 (4) | -3.68 (5) |
| **Gamma** | 1 | -3.87 (1) | -1.93 (1) | 0.03 (3) | 579.35 (2) | 4.15 (2) | -3.87 (1) |
| **Gaussian** | 0 | -3.23 (11) | -1.61 (9) | 0.09 (9) | 3578.20 (9) | 26.56 (9) | -3.23 (11) |
| **Power Law** | 0 | 0.245 (14) | 0.122 (12) | 0.33 (12) | 28021 (13) | 154.68 (12) | 0.245 (14) |
| **Maxwell** | 0 | -2.24 (13) | -1.12 (11) |  |  |  | -2.24 (13) |
| **Exponential** | 0 | -3.68 (5) | -1.84 (4) | 0.07 (7) | 3122.80 (8) | 15.79 (6) | -3.68 (5) |
| **Gen. Pareto** | 0 | -3.78 (4) | -1.89 (3) | 0.01 (1) | 6645.50 (10) |  | -3.78 (4) |
| **Fatigue** |  | -3.67 (6) | -1.83 (5) | 0.09 (9) | 2940.10 (7) | 16.57 (7) | -3.67 (6) |
| **Nakagami** |  | -3.84 (3) | -1.92 (2) |  |  |  | -3.84 (3) |
| **Weibull** |  | -3.86 (2) | -1.93 (1) | 0.03 (2) | 395.88 (1) | 2.94 (1) | -3.86 (2) |
| **Loglogistic** |  | -3.67 (6) | -1.83 (5) | 0.07 (8) | 1817.1 (6) | 20.05 (8) | -3.67 (6) |
| **T-location**  **scale** |  | -3.27 (9) | -1.63 (8) |  |  |  | -3.27 (9) |
| **Extreme Value** |  | -2.37 (12) | -1.18 (10) | 0.04 (4) | 863.43 (4) | 7.40 (3) | -2.37 (12) |
| **Inv. Gaussian** |  | -3.53 (7) | -1.76 (6) | 0.11 (10) | 8931.40 (11) | 46.51 (10) | -3.53 (7) |
| **Logistic** |  | -3.26 (10) | -1.63 (8) | 0.06 (6) | 1468.30 (5) | 11.74 (5) | -3.26 (10) |
| **Rayleigh** |  | -3.34 (8) | -1.67 (7) | 0.14 (11) | 10948.0 (12) | 61.20 (11) | -3.34 (8) |
| **Rician** |  | -3.34 (8) | -1.67 (7) |  |  |  | -3.34 (8) |

**Table S2.** **Extended Speed Fit Statistics.** Table shows the Akaike information criterion evidence ratio (AIC E), applied to first 7 rows only; the corrected Akaike information criterion (AICc); negative log-likelihood (nlogl), Kolmogorov-Smirnov (KS), Anderson-Darling (AD), chi-squared (χ^2^), and Bayesian information criterion (BIC). Score ranking is in parentheses. Differences in BIC and AICc scores are less than 1:10^3^ of the AICc score.
