# Supplementary material for: Persistence and Adaptation in Immunity: T Cells Balance the Extent and Thoroughness of Search
Source: PLoS Comput Biol. 2016 Mar 18;12(3):e1004818. doi: 10.1371/journal.pcbi.1004818 (PMC4798282; doi:10.1371/journal.pcbi.1004818)
Supplement: S3 Table — The lognormal probability distribution is still the best fit when steps are calculated using a 30° rather than 15° threshold. Compare to Table 1 in the main text. (DOCX) [file pcbi.1004818.s018.docx]

**Supplemental Table 3**

| **Distribution** | **-log Likelihood (×10^5^)** | **MLE Parameters** | |
| --- | --- | --- | --- |
| **Lognormal** | 4.89 | μ = 0.52 | σ = 1.00 |
| **Gaussian** | 6.53 | μ = 2.75 | σ = 3.24 |
| **Maxwell** | 8.40 | a = 6.00 |  |
| **Power Law** | 8.05 | α = 1.19 |  |

**Table S3.** **Maximum likelihood estimated parameters and associated likelihood scores for steps calculated using a 30° threshold.** The lognormal probability distribution is still the best fit when steps are calculated using a 30° rather than 15° threshold. Compare to Table 1 in the main text.
